# Maximal Segmental Score Method for Localizing Recessive Disease Variants Based on Sequence Data

**DOI:** 10.3389/fgene.2020.00555

**Published:** 2020-06-12

**Authors:** Ai-Ru Hsieh, Jia Jyun Sie, Chien Ching Chang, Jurg Ott, Ie-Bin Lian, Cathy S. J. Fann

**Affiliations:** ^1^Department of Statistics, Tamkang University, New Taipei, Taiwan; ^2^Graduate Institute of Statistics and Information Science, National Changhua University of Education, Changhua, Taiwan; ^3^Institute of Biomedical Sciences, Academia Sinica, Taipei, Taiwan; ^4^Laboratory of Statistical Genetics, Rockefeller University, New York, NY, United States

**Keywords:** whole-genome sequencing, rare disease, autosomal recessive disease, maximal segmental score, ALSPAC

## Abstract

**Background:**

Due to the affordability of whole-genome sequencing, the genetic association design can now address rare diseases. However, some common statistical association methods only consider homozygosity mapping and need several criteria, such as sliding windows of a given size and statistical significance threshold setting, such as *P*-value < 0.05 to achieve good power in rare disease association detection.

**Methods:**

Our region-specific method, called expanded maximal segmental score (eMSS), converts *p*-values into continuous scores based on the maximal segmental score (MSS) (Lin et al., 2014) for detecting disease-associated segments. Our eMSS considers the whole genome sequence data, not only regions of homozygosity in candidate genes. Unlike sliding window methods of a given size, eMSS does not need predetermined parameters, such as window size or minimum or maximum number of SNPs in a segment. The performance of eMSS was evaluated by simulations and real data analysis for autosomal recessive diseases multiple intestinal atresia (MIA) and osteogenesis imperfecta (OI), where the number of cases is extremely small. For the real data, the results by eMSS were compared with a state-of-the-art method, HDR-del (Imai et al., 2016).

**Results:**

Our simulation results show that eMSS had higher power as the number of non-causal haplotype blocks decreased. The type I error for eMSS under different scenarios was well controlled, *p* < 0.05. For our observed data, the bone morphogenetic protein 1 (*BMP1*) gene on chromosome 8, the Violaxanthin de-epoxidase-related chloroplast (*VDR*) gene on chromosome 12 associated with OI, and the tetratricopeptide repeat domain 7A (*TTC7A*) gene on chromosome 2 associated with MIA have previously been identified as harboring the relevant pathogenic mutations.

**Conclusions:**

When compared to HDR-del, our eMSS is powerful in analyzing even small numbers of recessive cases, and the results show that the method can further reduce numbers of candidate variants to a very small set of susceptibility pathogenic variants underlying OI and MIA. When we conduct whole-genome sequence analysis, eMSS used 3/5 the computation time of HDR-del. Without additional parameters needing to be set in the segment detection, the computational burden for eMSS is lower compared with that in other region-specific approaches.

## Introduction

In thousands of patients with rare diseases, whole-genome sequencing has been used to identify new causes of disease, which can help us increase our understanding of biological processes and improve clinical care. Because DNA sequencing is affordable, the genetic association design can now address diseases affecting fewer than 1,000 people. However, the statistical association methods that have been used so far to detect disease-related loci need several criteria to achieve good power in association detection, especially when the number of individuals with a rare disease is small. Compared to common diseases in the general population, rare diseases are low-prevalence health conditions that affect only a few individuals. Rare diseases include rare cancers, genetic diseases, and degenerative diseases. Among them, 80% of rare diseases are influenced by genetic effects (Perez, 2010; Valdez et al., 2016; The Lancet Diabetes Endocrinology, 2019). In situations in which the number of cases is very small (say, <5), traditional detection methods, such as logistic regression, may not be applicable, and non-parametric methods usually have low power. New statistical methods need to be developed to address such situations.

A single-marker association test may be inefficient when each genetic marker contributes only a small amount of association to a disease trait/phenotype. More powerful methods are region-specific tests, which take into account information on the heterogeneous genetic backgrounds of adjacent markers due to differences in allele frequencies and marker characteristics (Lin et al., 2012). Scan statistics with an exponential distribution of marker positions have been used to identify causal chromosomal regions using biological organization of single nucleotide polymorphism (SNP) data (Sun et al., 2009). For high-density association studies, the distribution assumption of scan statistics needs to be verified. Most sliding window methods assume a fixed or variable window size and then move windows along a sequence to evaluate the combined effects of markers within each window (Yang et al., 2006; Tang et al., 2009; Liu et al., 2011; Imai et al., 2016; Imai-Okazaki et al., 2017). HDR (Imai et al., 2016) and HDR-del (Imai-Okazaki et al., 2017) are two recently developed sliding window methods based on the Hamming distance for prioritizing variants in exome sequencing or deletions in exome sequencing, respectively. They map homozygosity regions longer than 1 Mb and calculate the “difference” between a case and control individual using the Hamming distance ratio (HDR) over all candidate exome sequences or deletions in exome sequencing.

For set limits on window size, Lin et al. (2012) provide a discrete scoring system, which is our previously developed region-specific test method not restricted by window size. These authors apply the concepts of a basic local alignment search tool (BLAST) (Altschul et al., 1990) and the maximal segmental score (MSS) method (Karlin and Altschul, 1990; Karlin and Dembo, 1992) in genome-wide association studies (GWAS). However, our previous study proposed using MSS to replace the discrete scoring system (Lin et al., 2012) with a continuous scoring system and apply it to detecting rare and common variants (Lin et al., 2014). In our previous MSS study, Lin et al. (2014) mentioned that it would be more appropriate to use a more general continuous score if a large case–control data set and effective computational power were available.

Autosomal recessive (AR) disorders usually occur in only one generation, so genetic linkage analysis is unlikely to be powerful. In previous AR studies, Imai et al. (2015, 2016) looked for differences in homozygosity patterns, i.e., homozygosity mapping (HM), around candidate variants between individual cases and individual controls and expected these differences in pathogenic variants to be greater than random candidate variants. The HM method is a common method for mapping AR diseases in consanguineous families. In most studies, applications for multipoint linkage analysis is to identify genomic regions associated with diseases (Seelow et al., 2009). However, these studies are neither suitable for very large families nor to accommodate tens of thousands of SNPs.

Here, we develop a novel region-specific method for the whole sequence called eMSS; it expands our previous continuous version of the MSS method (Lin et al., 2014) and is used to analyze disease-related segments in AR diseases detected by unrelated individuals. Our method expands previous HM methods from a candidate gene approach to a whole-genome sequence and works without sliding windows of a given size and additional parameters, such as minimum or maximum numbers of SNPs in a segment and minimum length of a segment. In this study, we address performance of our new method by evaluating results from simulations. We also compare sequence-variant segments between affected individuals from a Québec data set (Imai et al., 2015) and from a Pakistani data set (Kausar et al., 2018) and control individuals from the Avon Longitudinal Study of Parents and Children (ALSPAC) data sets (Boyd et al., 2013; Fraser et al., 2013). That is, our method compares single (or a few) affected individuals with a large number of control individuals. For any two individuals, we want to measure the discrepancy between their two variant arrays by a distance measurement. In our study, we analyze AR diseases, i.e., multiple intestinal atresia (MIA) and osteogenesis imperfecta (OI), in which pathogenic variants had been identified. Our region-based method successfully detects disease-causing segments in our simulation analysis and two AR disease data sets. The eMSS is likely to be powerful even with a very small number of observations and can further reduce variants to a very small set of susceptibility pathogenic variants.

## Materials and Methods

In short, the proposed eMSS converts any sequence of marker-wise testing results into a visualized MSS plot in which most susceptibility regions of markers can easily be identified. It comprises the following steps:

(1) To calculate marker-wise *p*-values on testing the difference between case and control groups.
(2) To transform *p*-values into appropriate scores.
(3) To convert the sequence of scores into an MSS plot and identify the region with maximal segmental score (MSS region).
(4) For subsequences in the above MSS region, repeat step 3 to search for the second MSS region, etc.

### Calculating Marker-Wise *p*-Values

There are many ways for testing marker-specific differences between case and control groups, and for our interest in rare diseases with extremely small numbers of cases, we use the following pairing scheme developed by Imai et al. (2016) as an example.

For each individual, the genotypes at a given site are coded *G* = 0 (REF/REF), 1 (REF/ALT or ALT/REF), or 2 (ALT/ALT). Imai et al. (2016) then reassigned the case and control groups into a “case–control” group and a “control–control group.” The case–control group comprised all possible case–control pairs, and the control–control group comprised all possible pairs among the controls. In the situation of a single case versus *m* controls, as illustrated in Imai et al. (2016), the case–control group consists of *m* case–control pairs, and the control–control group comprises *m*(*m* − *1*)*/2* control–control pairs. For a prespecified region width, they calculate a Hamming distance ratio (HDR) of the markers within it and obtain the *t* statistics on the difference of HDR between case–control and control–control groups. They show that the method is efficient to identify disease-related markers for rare diseases (Imai et al., 2015).

For illustration, we simplify the HDR method by considering only the smallest width of region that contains only one marker. Let (G1, G2)^*k*^ represent the two genotypes in the *k*th pair of individuals, where *k* ranges from 1 through *m* or *m*(*m* − *1*)*/2* for case–control or control–control pairs, respectively. The dissimilarity between the two individuals in a pair is then measured by the absolute difference, | G1 − G2|. For example, for a genotype pair of (1, 2) = (REF/ALT, ALT/ALT), the dissimilarity is |1 − 2| = 1.

If a variant is disease causing, the mean dissimilarity in case–control pairs is expected to be greater than that in control–control pairs, i.e., we use a one-sided, two-sample *t* statistic to test the following hypothesis:

H:0μ=case-controlμcontrolcontrol-

against

H:1μ>case-controlμcontrolcontrol-

The motivation for our approach is that we expect a larger “distance” between individual cases and individual controls for DNA fragments containing pathogenic variants than for random DNA fragments.

### Scoring System of *p*-Values Based on Fisher Transformation

To take into account the markers flanking each SNP, we develop a scoring system based on our previous continuous version of the MSS method. MSS was proposed by Karlin and Dembo (1992) and was originally applied in BLAST (Altschul et al., 1990), a widely used bioinformatics tool for protein or DNA sequence alignment and searching. It considers a sequence of discrete random variables *Y*_1_, …, *Y*_*n*_, which represent the dissimilarity of *n* markers between the DNA sequences of two individuals. As the simplest example, *Y*_*i*_ = *1* if, at marker *i*, both sequences carry the same genotype; otherwise, *Y*_*i*_ = −*1*. Note that a necessary condition for MSS to work is *E*[*Y*_*i*_] < 0, i.e., *Y*_*i*_ should more likely be negative than positive in the sequence.

In our application, we replace the discrete *Y*_*i*_ by a continuous score, which is a monotone function of *P*_*i*_ (the *p*-value), where *P*_*i*_ is obtained from a two-sample *t*-test on testing the difference between the case–control and control–control groups at marker *i* in the sequence.

To smooth the wiggly plot based on original *p*-values and make the MSS plot more visually friendly, we employ the Fisher transformation, −2ln(*P*_*i*_), which transforms a uniformly distributed *p*-value into a chi-square distribution with 2 degrees of freedom. Furthermore, to allow for the MSS condition, *E*[*Y*_*i*_] < 0, we use the left-censored *P*_*i*_ truncated by its 10th percentile (denoted as *P*_*c*_) to avoid infinity from extremely small *P*_*i*_, i.e.,

$$P_{\text{i}}=\left\{ \begin{array}{c} P_{\text{c},}\,i\,f\,\,P_{\text{i}}\leq P_{\text{c}}\\ P_{\text{i},}\,i\,f\,P_{\text{i}}>P_{\text{c}} \end{array}\right.$$

and define the score as

*Y*_i_ = −2 ln (*P*_i_) − *P*_f_, *i* = *1*,…, *n* markers,

where *P*_f_ is set to be $f\times \left(-\sum_{i=1}^{n}2\,\text{ln}\,P_{\text{i}}\right)/\text{n}$, with *f* > 1 to assure a negative mean of *Y*_*i*_ (*E*[*Y*_*i*_] < 0). The choice of *f* is analogous to the rotation parameters in factor analyses, in which some *f* can make the display of the MSS plot easier to identify susceptibility regions of markers. In our applications, we found that *f* = 1.2–1.5 has the best visualization. We illustrate it by simple examples after the description of the MSS procedure.

The MSS procedure includes the following steps:

(1) Calculating the partial sum of the sequence of marker scores

The single marker scores [*Y*_1_, …, *Y*_*n*_] are aggregated in a sequential manner to form a sequence of partial sum scores [*U*_0_, *U*_1_, …, *U*_*n*_], where *U*_0_ = 0 is the initial partial sum score, and $U_{\text{m}}=\sum_{i=1}^{m}Y_{\text{i}}$, where *m* = 1, 2, *…*, *n*. *U*_m_ is the partial sum up to the *m*th marker in the sequence. The partial sum scores *U*_m_ as a function of the single marker scores *Y*_*i*_ are increasing or decreasing with *i* because *Y*_*i*_ can be negative.

(2) Identifying ladder points and subsequences

**FIGURE 1:**
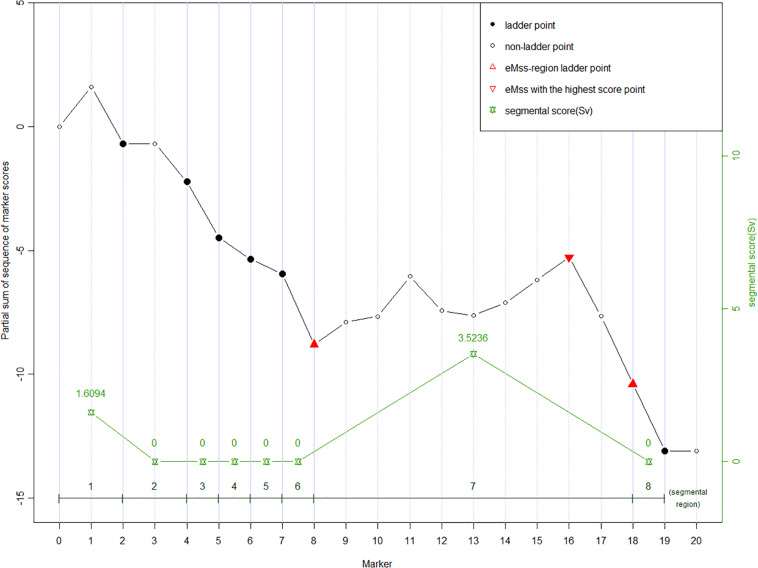
An example of partial sum scores for 20 SNPs. Ladder points in solid dots (2nd, 4th to 8th, 18th, and 19th SNP) forming eight segmental regions are the markers with new record lows of the partial sum scores, which are lower than all scores on their left, respectively. The ladder points partition the curve into the up-climbing intervals (e.g., [0 to 2nd], [2nd to 4th], and [8th to 18th]), which indicate the cluster of neighboring significant SNPs and the declining regions (the rest insignificant intervals). Segmental score (Sv, green stars) is the vertical height of each interval, and the highest Sv is assumed to be the most susceptive region. In eMSS, some minor number of insignificant SNPs within the susceptive region are tolerable, e.g., markers 12 and 13.

Given a partial sum score sequence [*U*_0_, *U*_1_, …, *U*_*n*_], ladder points are defined as *J*_0_ = 0, *J*_v_ = *min*_j_ {*j* : *j* ≥ *J*_v + 1_ + 1, *U*_j_ − *U*_*J*_v + 1__ < 0}, *v* = 1, 2, …, *n*_*l*_, where *n*_*l*_ = the number of ladder points (Karlin and Dembo, 1992). Figure 1 shows an example of 20 SNP markers labeled ladder points (solid dots). As the calculation moves along the markers, the ladder points are at the new low points of the partial sum scores. Once the ladder points have been set along the marker sequence, regions defined by two adjacent ladder points form subsequences. As shown in Figure 1, there are eight ladder points, forming eight subsequences. The ladder points are the second, fourth to eighth, 18th, and 19th SNPs with low point partial sums *U*_2_, *U*_4_ to *U*_8_, *U*_18_, and *U*_19_, respectively. Note that a ladder point appears if the partial sum of a ladder point is less than the partial sum of the previous ladder point. In our study, the purpose of the ladder points is to form subsequences along the marker sequence in each of the subsequences. We can calculate the general segment score as shown in the next section. Using ladder points can help us find the region with the highest score, which may be the region associated with the disease. This also helps reducing the data dimension inherent in high-throughput data.

(3) Identifying the maximal segmental score

For each subsequence, the segmental score, *Sv*, is computed as the difference between the largest partial sum of a subsequence and the initial partial sum of the subsequence. The equation used to obtain the segmental score is as follows:

$$S_{\text{v}}=\max_{J_{v-1}\leq j<J_{\text{v}}}\left(U_{\text{j}}-U_{{\text{J}}_{v-1}}\right),$$

where *Sv* is a non-negative value defined as the difference between the largest partial sum and the initial partial sum value within the subsequence defined by two adjacent ladder points. In the example (Figure 1), the *Sv* values for the eight subsequences are (*U*_2_ − 0), 0, 0, 0, 0, 0, (*U*_18_ − *U*_8_), 0. A zero score denotes no increase between two adjacent ladder points. The larger the score, the more likely the target SNP is in the segment. The maximum segmental score for the whole sequence is recognized as eMSS = max [*S*_1_, *S*_2_, …]. In the example shown in Figure 1, eMSS is given by (*U*_18_ − *U*_8_). The statistical significance of eMSS is assessed by permutation as shown below.

(4) Permutation test

To test the null hypothesis of no disease genes, the *p*-value of the eMSS is estimated by permutation. In order to derive the null distribution of eMSS while preserving the genotype data structure, we generate *m* replicates by randomly shuffling the case–control status of all individuals and then carrying out the above described steps each time. The observed eMSS is then compared to the null distribution to derive the empirical *p*-value, which is defined as the proportion of permuted eMSS at least as large as the observed eMSS. A significance level of 0.05 was used here.

(5) Varying *f* for better visualization of MSS plots

The idea of MSS is to highlight the region between two adjacent ladder points that represent the difference between the two groups (case–control and control–control) in this region. The segmental score is the maximum height in this region, and the region with highest MSS is considered to be the most likely to contain the disease-related markers than other regions. The ideal situation is that the MSS region occurs with as few markers as possible so that the disease-related genes can easily be identified. If *f* is small, say *f* = 1, the region with MSS tends to be too useless, i.e., the region is nearly half the whole sequence and is not helpful for identifying the small number of target markers. Supplementary Figure S8 illustrates an MSS plot under different *f*. In Supplementary Figure S8A with *f* = 1, the region is nearly half the whole sequence, which is not helpful for identifying the small number of target markers. On the other hand, if *f* is large, say *f* = 1.25, then the *Y*_*i*_s are mostly negative, and the MSS plot tends to be steeply decreasing either without any positive segmental score or non-significant MSS (Supplementary Figure S8B). An ideal plot is with large MSS within a narrow region, i.e., large MSS with small *W*, where *W* is the number of markers within the MSS region bounded by two adjacent ladder points. We, therefore, consider the following strategy: equally dividing 1 to 1.25 into 10 intervals, creating an MSS plot with *f* = 1, 1.025, 1.05, …, 1.225, and 1.25, and choosing the one with maximal MSS/W as the optimal plot. Supplementary Figure S8C shows that *f* = 1.14 is optimal in the experimental data.

## Results

### Simulation

In our simulation study, we used the simulation software SNaP (Nothnagel, 2002) to generate SNPs in a case–control association study of one case and 32 controls. We considered the four factors described below when generating genotype data.

(1) Number of haplotype blocks: Gabriel et al. (2002) found that only two or three markers were sufficient to detect regions as blocks. Based on this information, we considered numbers of blocks at three levels: high density (H, 100 blocks, including 302 SNPs), moderate density (M, 36 blocks, including 110 SNPs), and low density (L, 15 blocks, including 47 SNPs);
(2) Haplotype frequency: Gabriel et al. (2002) also observed that most blocks contained only three to five haplotypes, and these major haplotypes can provide 90% of the information for a given block. Based on the above information, we set two levels of haplotype frequencies: extreme (E), that is, one of the major haplotypes has a frequency ≥ 0.8, and non-extreme (nE), that is, one of the major haplotypes has a frequency < 0.6. For the E haplotype frequency pattern, we assumed that, of the three haplotypes, the major haplotype has a frequency of 0.8 and the other two haplotypes have a frequency of 0.1. For the nE haplotype frequency pattern, we assumed that, of the three haplotypes, the major haplotype has a frequency 0.6 and the other two haplotypes have a frequency of 0.2.
(3) Allele frequency (AF): The allele frequency of a causal variant at AF = 0.01 and AF = 0.001.
(4) Number of cases and controls: one case versus 32 controls, one case versus 50 controls, and three cases versus 32 controls.

First, the simulation results showed that eMSS had higher power and lower type I error in low-density haplotype blocks (15 blocks, including 47 SNPs) than in high-density (100 blocks, including 302 SNPs) haplotype blocks (77.8% vs. 96.2% in the E haplotype frequency pattern, Table 1). The statistical power to identify pathogenic regions decreased as the number of non-causal haplotype blocks, i.e., non-causal SNPs, increased. Second, eMSS had higher power in the E haplotype frequency pattern than in the nE one. In the E haplotype frequency pattern, the power was 96.2% for the L block and 79.7% for the H block (Table 1). In the nE haplotype frequency pattern, the power was 93.4% for the L block and 65.3% for the H block (Table 1). However, for the H block and nE haplotype frequency pattern (the worst scenario), eMSS found a 30.9% decrease in power, down to 65.3%. Third, the allele frequency of a causal variant would not affect the performance of eMSS. The power and type I error at AF = 0.01 and AF = 0.001 in eMSS was quite similar. Fourth, increasing the number of controls would not increase the statistical power, but increasing the number of cases would achieve the statistical power to 86% for the H block and nE haplotype frequency pattern (the worst scenario) (Supplementary Table S6).

**TABLE 1:** Power and type I error comparison with differing scenarios in our eMSS calculations under the two-sample *t*-test.

**Power (Type I error)**	**Number of haplotype blocks**							
**Haplotype frequency**	**High density 100 Blocks (including 302 SNPs)**				**Moderate density 36 Blocks (including 110 SNPs)**		**Low density 15 Blocks (including 47 SNPs)**	
	**AF^3^ = 0.01**		**AF = 0.001**		**AF = 0.01**	**AF = 0.001**	**AF = 0.01**	**AF = 0.001**
	**eMSS**	**HDR-del^4^**	**eMSS**	**HDR-del^4^**	**eMSS**	**eMSS**	**eMSS**	**eMSS**
Extreme^1^	76.7% (1.5%)	76% (7%)	77.8% (1.2%)	75% (8%)	80.7% (2.5%)	79.7% (1.6%)	96.2% (1.9%)	96.2% (0.6%)
Non-extreme^2^	63.5% (3.5%)	60% (7%)	65.1% (3.4%)	64% (6%)	68.3% (3.5%)	65.3% (3.0%)	91.4% (4.9%)	93.4% (0.2%)

*^1^The frequency of one major haplotype was ≥0.8. ^2^The frequency of one major haplotype was <0.6. ^3^The allele frequency of one causal variant. ^4^HDR-del is suitable for longer sequence, so HDR-del is not applicable for the other scenarios.*

HDR-del is a web-based method, and the parameters need to be fine-tuned. The power and type 1 error for the first and second hundred in our simulation are very similar, so we use the average of them in Table 1. HDR-del considers runs of homozygosity (ROHs) longer than 1 Mb (Imai-Okazaki et al., 2017). Thus, we only made power and type I error comparisons under the scenario with high-density haplotype blocks (100 blocks, including 302 SNPs). It seems that eMSS had higher power and lower type I error than HDR-del under the scenario with high-density haplotype blocks [power (type I error) = 77.8% (1.2%) for eMSS vs. 75% (8%) for HDR-del in the E haplotype frequency pattern, Table 1].

### Real Data Analyses—Québec

As a proof of concept for the study, we applied our approach to MIA and OI patients for which the AR disease-related regions had previously been detected and published (Bernard et al., 2011; Tetreault et al., 2011; Srour et al., 2012; Fahiminiya et al., 2015). For MIA, a pathogenic variant on chromosome 2 in the tetratricopeptide repeat domain 7A (*TTC7A*) gene (Samuels et al., 2013) was found in three affected individuals, i.e., patients F1, F4, F6. For OI, a pathogenic variant on chromosome 8 in the bone morphogenetic protein 1 (*BMP1*) gene (Fahiminiya et al., 2015) was observed in an affected individual, i.e., patient OI. patients F1, F4, F6, and OI are members of the French–Canadian population of Québec, which has about 8,500 French settlers who immigrated more than 300 years ago (Laberge et al., 2005). Because of the limitations of data set ethical approval, control individuals cannot be of the same ethnic background as case individuals. Hence, we randomly selected 32 individuals as control individuals from the ALSPAC Cohort Project (Boyd et al., 2013; Fraser et al., 2013). All affected individuals had been exome-sequenced at McGill University and the Genome Québec Innovation Center, Montreal, Canada as detailed in publications (Imai et al., 2015, 2016). In the ALSPAC study, researchers sequenced 1927 individual exomes to 80× coverage and found 842,646 SNVs and 6067 indels. The ALSPAC study website contains details of all data available through a fully searchable data dictionary and variable search tool^1^.

Results obtained by our eMSS analysis show that we were able to identify the OI disease variants to be in the top three of sequence segments ranked on chromosome 8 (Table 2) and MIA disease variants on chromosome 2 (Table 2). For OI analysis, there were 5,628 SNPs was used to run eMSS on chromosome 8 for the intersection of two sets ALSPAC and OI. In the data set of one OI and 32 ALSPAC controls, the length of the segment ranked 2 detected by our eMSS method was 3,475 kb on chromosome 8 (294 SNPs spanning 3,475 KB, *P* = 0.015). In refinement analysis (i.e., eMSS refinement in Table 2), eMSS was rerun using only the causal region from the first time (i.e., 294 SNP spanning 3,475 KB) in hope of further narrowing down the region size. The result from the second run showed that the final causal region included 10 SNPs spanning 30.145 KB (*P* = 0.015, Table 2 and Supplementary Figure S1). The sequence segment (chr8: 22,022,749–22,052,894 bp) comprised the following gene: *BMP1*. That segment ranked 1 (best) on chromosome 8 for OI associated analysis using the ALSPAC data set.

**TABLE 2:** Ranking of known pathogenic variants of the Québec data set used in our eMSS method.

				**eMSS**				**eMSS refinement**			
**Patient**	**Disease**	**Chr**	**Num**	**Position**	***m***	**Rank**	***P*-value**	**Position**	***m***	***P*-value**	**Gene**
OI	OI	8	5,628	20,884,737–24,359,279	294	2	0.015	22,022,749–22,052,894 (30.145 kb)	10	0.015	*BMP1*
F1	MIA	2	8,465	45,205,547–71,635,931	744	2	0.015	47,251,634–47,256,618 (4.984 kb)	4	0.030	*TTC7A*
F4	MIA	2	7,380	39,108,773–55,528,514	378	1	0.015	47,133,330–47,273,668 (140.358 kb)	11	0.015	*MCFD2, TTC7A*
F6	MIA	2	7,562	45,171,842–85,059,227	872	1	0.015	47,133,330–47,273,668 (140.358 kb)	8	0.015	*MCFD2, TTC7A*
F1 + F4 + F6	MIA	2	3,484	38,916,906–68,676,008	386	1	0.002	47,133,330–47,277,043 (143.713 kb)	9	0.002	*MCFD2, TTC7A*

*OI, osteogenesis imperfecta; MIA, multiple intestinal atresia; Chr, chromosome; num, number of variants for the intersection of two sets ALSPAC and affected family; rank, order of segmental score among sequence segments on chromosome 2 and 8, respectively; m, number of variants in the eMSS region.*

For MIA analysis, there were 3,484 SNPs on chromosome 2 for the intersection of two sets ALSPAC and three MIA cases (patients F1, F4, and F6). In the data set of three MIA cases and 32 ALSPAC controls, the length of the most significant sequence segment detected by the eMSS method was 29,759 kb (386 SNPs, *P* = 0.002). In the eMSS refinement, the sequence segment extended over 143 kb on chromosome 2 (9 SNPs, *P* = 0.002) (Table 2 and Supplementary Figure S2). This sequence segment comprised the following genes: multiple coagulation factor deficiency 2 (*MCFD2*) and tetratricopeptide repeat domain 7A (*TTC7A*). Using the ALSPAC data set, that segment ranks first on chromosome 2 of the MIA correlation analysis.

As the OI study works with one affected individual versus 32 control individuals, the three individuals in MIA (patients F1, F4, and F6) were also considered separately in our study. The most significant sequence segment detected by the eMSS method in all three MIA cases considered together, the sequence segment (29,759 kb; 385 SNPs, *P* = 0.002 in all three MIA cases considered together) was also detected by patients F1 (26,430 kb; 744 SNPs; *P* = 0.015), F4 (16,419 kb; 378 SNPs; *P* = 0.015), and F6 (39,887 kb; 872 SNPs; *P* = 0.015), separately. In the eMSS refinement for the patient F1, the sequence segment ranged over 4 kb on chromosome 2 (4 SNPs, *P* = 0.030) (Table 2 and Supplementary Figure S3). Thus, patients F1, F4, and F6 detect the same pathogenic region for MIA with patient F1 furnishing the shortest region (Table 2 and Supplementary Figures S4, S5).

Mutations had been identified (1) in the *BMP1* gene on chromosome 8 associated with recessive OI and (2) in the *TTC7A* gene on chromosome 2 associated with recessive MIA (Bernard et al., 2011; Tetreault et al., 2011; Fahiminiya et al., 2015). Thus, we showed significantly larger distances between case individuals and control individuals for pathogenic variants than random variants. Furthermore, we successfully narrowed down the segment detected in our OI- and MIA-associated analysis. Supplementary Table S1 shows the three top-ranked susceptibility regions across 22 chromosomes used in our eMSS calculations.

To compare the ability of detecting susceptibility regions between our eMSS method and other region-specific methods, we applied the HDR-del (Imai-Okazaki et al., 2017) approach using the same vcf files for case and control individuals as in our eMSS method. HDR-del defines ROH as a sequence of homozygous variants bounded by one or more single-nucleotide heterozygous variants. HDR-del considers ROHs longer than 1 Mb (Imai-Okazaki et al., 2017). As shown in Supplementary Table S3, out of 213–375 candidate variants, HDR-del considered ROHs at least 1 Mb long and succeeded in detecting the pathogenic regions in OI (chr8:21,471,941–23,622,382, rank = 32, *p* = 0.0303) and F6 (chr2: 45,171,842–52,799,698, rank = 7, *p* = 0.0303) patients. When considering ROHs of length between ±1.5 Mb of the lengths of the pathogenic regions, out of 4–345 candidate variants, HDR-del successfully narrowed down pathogenic variants to be ranked 2 (for F6) through 26 (for OI) (Supplementary Table S3). When considering ROHs of length between ±0.5 Mb of the lengths of the pathogenic regions, out of 2–110 candidate variants, HDR-del successfully narrowed down pathogenic variants to be ranked 1 (for F6) through 7 (for OI) (Supplementary Table S3).

Except that eMSS flags shorter causal chromosomal regions, eMSS succeeded in detecting a pathogenic region for OI and MIA (F1, F4, and F6). However, we have fine-tuned the parameter settings, such as window size, but HDR-del only succeeded in detecting OI and F6.

### Authentic Data Analyses—Pakistani

We also applied eMSS to other real data from Chinute, Pakistan (Kausar et al., 2018). Blood samples were drawn from two related individuals (patients III-5 and III-15) in the same family (Kausar et al., 2018) with a reported *VDR* mutation responsible for OI.

Because the OI cases are from the same family, our study considered two OI cases (patients III-5 and III-15) separately versus 32 control individuals from the ALSPAC (Boyd et al., 2013; Fraser et al., 2013) data set. For OI analysis, there were around 2,000 SNPs (2,229 for patient III-5 and 2,022 for patient III-15, Table 3) on chromosome 12 for the intersection of two sets ALSPAC and OI. The significant sequence segment was detected by patient III-5 (20,925 kb; 196 SNPs; *P* = 0.015, Table 3), and patient III-15 (5,534 kb; 146 SNPs; *P* = 0.015, Table 3), separately. In the eMSS refinement for patient III-5, the sequence segment ranged over 610 kb on chromosome 12 (8 SNPs, *P* = 0.015, Table 3 and Supplementary Figure S6), and the sequence segment ranged over 611 kb on chromosome 12 (9 SNPs, *P* = 0.015, Table 3 and Supplementary Figure S7) for patient III-15. Thus, patients III-5 and III-15 detect the same pathogenic region for OI with patient III-5 furnishing the shortest region (Table 3 and Supplementary Figures S6, S7). Mutations had been identified in the *VDR* gene on chromosome 12 associated with recessive OI (Bernard et al., 2011; Tetreault et al., 2011; Fahiminiya et al., 2015). Furthermore, we successfully narrowed down the segment detected in our OI-associated analysis. Supplementary Table S2 shows the three top-ranked susceptibility regions across 22 chromosomes used in our eMSS calculations.

**TABLE 3:** Ranking of known pathogenic variants of the Pakistani data set to the OI disease used in our eMSS method.

			**eMSS**				**eMSS refinement**			
**Patient**	**Chr**	**Num**	**Position**	***m***	**Rank**	***P*-value**	**Position**	***m***	***P*-value**	**Gene**
III-5	12	2,229	31,545,381–52,470,979	196	2	0.015	48,272,895–48,883,020 (610.125 kb)	8	0.015	*VDR, SENP1, ASB8, H1FNT*
III-15	12	2,022	47,178,307–52,713,088	146	2	0.015	48,272,895–48,884,535 (611.640 kb)	9	0.015	*VDR, SENP1, ASB8, H1FNT*

*OI, osteogenesis imperfecta; Chr, chromosome; num, number of variants for the intersection of two sets ALSPAC and affected family; rank, order of segmental score among sequence segments on chromosome 2 and 8, respectively; m, number of variants in the eMSS region.*

We also applied the HDR-del (Imai-Okazaki et al., 2017) approach using the same vcf files for case and control individuals as in our eMSS method to compare the ability of identifying susceptibility regions between eMSS and other region-specific methods. As shown in Supplementary Table S4, out of 103–122 candidate variants, HDR-del considered ROHs at least 7 Mb long and succeeded in detecting the pathogenic regions in patient III-5 (chr12: 34,175,508–52,404,618, rank = 5, *p* = 0.0303, Supplementary Table S4) and patient III-15 (chr12: 45,410,075–52,602,013, rank = 16, *p* = 0.0303, Supplementary Table S4). When considering ROHs of lengths between ±1.5 Mb of the lengths of the pathogenic regions, out of 2–11 candidate variants, HDR-del successfully narrowed down pathogenic variants to be ranked 1 (for patient III-5 and patient III-15) (Supplementary Table S4). When considering ROHs of lengths between ±0.5 Mb of the lengths of the pathogenic regions, out of 2–4 candidate variants, HDR-del successfully narrowed down pathogenic variants to be ranked 1 (for patient III-5 and III-15) (Supplementary Table S4).

Although eMSS and HDR-del both successfully detected the causal chromosomal regions of III-5 and III-15, the precision of the two methods to determine the causal regions was not similar. In the eMSS refinement, the candidate causal region flagged in the eMSS can keep shrinking this region.

## Discussion

Our eMSS method converts *p*-values into continuous scores and does not need to set segment sizes. The eMSS has several advantages over existing HDR-based and MSS-based methods (Lian et al., 2008; Lin et al., 2012, 2014; Imai et al., 2015, 2016; Imai-Okazaki et al., 2017): (1) Our eMSS considers the whole genome, not only regions of homozygosity in candidate genes. (2) Most HDR-based and MSS-based approaches work with sliding windows of a given size and additional parameters, such as a minimum or maximum number of SNPs in a segment and minimum length of a segment, and our approach does not require such parameters. (3) Most MSS approaches have thresholds for statistical significance setting. These settings may or may not be optimal. (4) Since no additional parameters are set in the segment detection, eMSS reduces the computational burden of the region-specific approaches.

We drive the selection of ladder points by matching amino acid sequences based on similarity scores (such as BLAST) (Altschul et al., 1990; Karlin and Altschul, 1990; Karlin and Dembo, 1992). In our study, the purpose of ladder points is to form subsequences along the input marker sequence so that we can calculate eMSS in each of the smaller regions. Using ladder points helps us find the region with the highest score, which may be the associated region to identify the disease. This also helps to reduce the data dimensionality inherent in high-throughput data caused by sequence data.

In our simulation study, we also replace *P*_*i*_ in *Y*_*i*_ from a two-sample *t*-test to a simple *t*-test for a difference in case and control means. For a single case individual, by subtracting case value from each control, the case–control group has a mean equal to the difference between the control mean and the case. However, for the control–control group, since the subtractor and subtrahend are in random order, the mean of this group is not necessarily zero; therefore, the HDR *t* statistic does not wind down to a one-sample *t*-test. Actually our simulation had shown that the one-sample *t*-test has much lower power due to the small sample size. From our simulation result, in the L block and E haplotype frequency pattern, we found that eMSS with the *P*_*i*_ obtained from a one-sample *t*-test had much lower power (55.1%) compared to 96.2% from a two-sample *t*-test. Similarly, in the worst scenario (i.e., H block and nE haplotype frequency pattern), eMSS with the *P*_*i*_ obtained from a one-sample *t*-test showed a 59.8% decrease in power down to 5.5%. Moreover, eMSS with the *P*_*i*_ obtained from a one-sample *t*-test gave a slightly higher type I error. Especially at H block and nE haplotype frequency pattern, eMSS with the *P*_*i*_ obtained from a one-sample *t*-test had 11.5% type I error compared to 3.0% with the *P*_*i*_ obtained from a two-sample *t*-test (Table 1).

Here, we demonstrate that, by applying eMSS, we can detect the same pathogenic genes as those shown by Imai et al. (2015) and Kausar et al. (2018). However, HDR-del (Imai-Okazaki et al., 2017) also detected the same genes in our two patients, thus also confirming our approach. Our eMSS method does not require any prior assumptions or parameters. In contrast, HDR-del defined an ROH as a sequence of homozygous variants bounded by one or more single-nucleotide heterozygous variants (Imai-Okazaki et al., 2017), which requires the following prerequisite parameters, such as ROH distance and pathogenic regions. Furthermore, the range of pathogenic regions detected by our eMSS (ranged from 4 to 143 kb) are much shorter than those detected by HDR-del (ranged from 2.04 Mb through 11.17 Mb) in the Québec data set analysis. Of course, functional analysis is the ultimate proof, but it has not been available in our study. However, we are relieved that our eMSS method can reduce variants to very small segments of potential pathogenic variations. Our approach is very useful for clinicians who search out disease-causing genes for their case individuals and also for scientists who are trying to identify pathogenic variants through functional analysis. In our Québec and Pakistani data set analyses, we used ALSPAC as our control individuals. From ALSPAC as control individuals, the *BMP1* gene on chromosome 8 associated with OI, the *VDR* gene on chromosome 12 associated with OI, and the *TTC7A* gene on chromosome 2 associated with MIA could be successfully detected by eMSS (Tables 2, 3). It is also suitable for detecting associated segments with both common and rare variants.

Osteogenesis imperfecta is a genetic disease characterized by low bone mass, increased bone fragility, and recurrent fractures (Marini et al., 2017). Autosomal recessive OI patients are extremely rare, of which OI type XIII can be attributed to *BMP1* gene mutations. *BMP1* was first identified in 2012 (Asharani et al., 2012) as the disease-related gene of OI type XIII (OMIM 614856) in families with progressively deforming bones. The OI type IV is a clinical entity with autosomal dominant inheritance in type 1 collagen genes; collagen, type I, alpha 2 (*COL1A2*), and more rarely, collagen, type I, alpha 1 (*COL1A1*) point mutation or small deletion and short stature. Ayça et al. (2015) recalls the role of the *VDR* anomalies in the development of the diseases occurring with hereditary osteoporosis. The pathogenic region in *BMP1* and *VDR* can be detected successfully in our OI analysis. Our eMSS can analyze rare disease one-case studies and is very useful for detecting pathogenic regions related to specific disease subtypes.

For MIA, our data set involved three case individuals (F1, F4, and F6). We compared the results from consideration of three MIA case individuals together and the consideration of three MIA case individuals separately; we found the latter consideration in the refinement could efficiently narrow down the segment size (Table 2 and Supplementary Figures S1–S5). Therefore, our eMSS method is more efficient when there is lower variation in the analyzed data set.

In our Québec authentic data analysis, we combined 22 chromosomes in sequence data to detect segments potentially containing pathogenic variants via our eMSS method, but the most important sequence segment ranging from 20,884,737 to 25,042,515 bp on chromosome 8 cannot be detected in our OI analysis. One of the possible reasons is that gene density varies greatly among the chromosomes (Lander et al., 2001; Venter et al., 2001; Payseur and Nachman, 2002). For example, sequence data suggest that chromosome 19 has an average of 23 genes per Mb, and chromosome 4 has averages only six genes per Mb (Venter et al., 2001; Payseur and Nachman, 2002). If we used the one-of-a-kind *P*_*f*_ settings to detect associated segments across 22 chromosomes in a sequence, pathogenic variation information might be diluted. Hence, we suggest that researchers use our eMSS method separately by chromosomes.

## Conclusion

eMSS is based on association statistics (or *p*-values) not limited to a particular test. On the other hand, an appeal of *p*-value–based methods is that raw data is often not required. The eMSS avoids predefined thresholds or window sizes in identification of tentative association regions. On the contrary, HDR-del is limited to a particular test and parameter settings to achieve better detecting performance for causal regions.

Homozygosity Mapper (Pippucci et al., 2014) provides an online intuitive graphical interface that allows users to interactively analyze NGS data for homozygosity mapping. HomozygosityMapper is a sliding-window approach; we need to set the length of the window size before performing mapping. Generally, long ROH can be detected (ROH > 1.5 Mb) by HomozygosityMapper (Pippucci et al., 2014). HomozygosityMapper is better for longer sequences (Pippucci et al., 2014). Thus, we only made power comparison under the scenario with high-density haplotype blocks (100 blocks, including 302 SNPs, Supplementary Table S7). The results show that HomozygosityMapper had lower power than that obtained from eMSS and HDR-del (power = 77.8% for eMSS, it’s 75% for HDR-del and 44% for HomozygosityMapper in the E haplotype frequency pattern given AF = 0.001 shown in Table 1 and Supplementary Table S7). In this study, genotypes were simulated as 00, 01, and 11 under the VCF format. HomozygosityMapper screens all samples for blocks of homozygous genotypes in contiguous markers; that is, 00 and 11 are both considered to be homozygous (Pippucci et al., 2014). That might be one of the reasons that HomozygosityMapper had lower power than eMSS and HDR-del and high type I error in our simulation study. HDR-del is the other homozygosity mapping method, which prioritizes pathogenic chromosomal deletions based on Hamming distance in exome sequencing. HDR-del is also a sliding window approach, which can narrow down true disease-related chromosomal deletion regions by setting the window size. However, our eMSS method expands previous homozygous mapping from the candidate gene approach to the whole-genome sequence and can detect disease-related segments without setting a window size. Moreover, we found that the segments found by eMSS are much shorter than those found by HDR-del (Table 3 and Supplementary Table S2) in our authentic data analyses.

In Pakistani previously published results, Kausar et al. (2018) report that a Wnt family member 1 (*WNT1*) mutation is responsible for OI. However, there was only one SNP located in the *WNT1* on chromosome 12 for the intersection of two sets ALSPAC and Pakistani in our authentic data set analysis. Hence, eMSS cannot find significant segments located in the *WNT1* in our study. That is one of the limitations of using public data.

A limitation of our study is that eMSS requires control individuals in addition to a single or a few case individuals in our authentic data analyses. We used 32 control individuals as a compromise between cost and efficiency, which proved to be adequate for the given data. Applied to our case individuals and corresponding control individuals, the eMSS method narrowed down the length of the pathogenic sequence segment more than the previous region-specific method (Lin et al., 2014). Although based on few observations, these results are encouraging and demonstrate the potential of our approach. We implemented our program in R and make it available for free. It is easy to use and does not require expertise in computer science. The OI analysis contains one case and 32 controls; our eMSS performs whole-genome sequence analysis without a sliding window, thereby overcoming the computational burden of a genome scan due to the sliding window-based method. When we conduct whole-genome sequence analysis with Intel(R) Xeon(R) CPU E3-1230 V2 at 3.30 GHz (24 GB RAM), the computing time of the eMSS analysis was 6 min. On the other hand, HDR-del took 10 min. Hence, eMSS used 3/5 the computation time of HDR-del analysis. Our approach is computationally more efficient than HDR-based methods (Imai et al., 2016; Imai-Okazaki et al., 2017). As far as we know, there are no other pieces of software comparable to ours.

## Data Availability Statement

**Program Availability:** The software package containing the R source code and a detailed documentation are freely available for download at http://www.csjfann.ibms.sinica.edu.tw/eag/programlist/eMSS/eMSS.html. **Availability of Data and Material:** Source code developed for this project is available at http://www.csjfann.ibms.sinica.edu.tw/eag/programlist/eMSS/eMSS.html. Data to ALSPAC samples used in our manuscript have been deposited with the European Genome-phenome Archive (EGA), Wellcome Trust Genome Campus, Hinxton, Cambridge CB10 1SD, United Kingdom, with study accession number EGAD0000100740.

## Author Contributions

A-RH, JO, I-BL, and CF contributed to the statistical analysis and writing of the manuscript. JS and CC contributed to the statistical analysis.

## Conflict of Interest

The authors declare that the research was conducted in the absence of any commercial or financial relationships that could be construed as a potential conflict of interest.

## Footnotes

1 http://www.bristol.ac.uk/alspac/researchers/our-data/
