# Supplementary material for: Maximal Segmental Score Method for Localizing Recessive Disease Variants Based on Sequence Data
Source: Front Genet. 2020 Jun 12;11:555. doi: 10.3389/fgene.2020.00555 (PMC7325894; doi:10.3389/fgene.2020.00555)
Supplement: Supplementary file 1 [file Presentation_1.zip › Figure S1.DOCX]

Supplementary Table S1

All shortlisted candidate regions in each of the four patients (Patients OI, F1, F4, F6) used in our eMSS calculations

Patients OI.

| **chr** | **rank** | **eMSS** | **p-value** | **1^st^  eMSS refinement** | **p-value** | **2^nd^ eMSS refinement** | **p-value** | **Gene symbol** |
| --- | --- | --- | --- | --- | --- | --- | --- | --- |
| **1** | 1 | 156850112～230418647 | 0.01515 | 156875985～157029984 | 0.01515 | 156907081～156939067 | 0.0303 | *ARHGEF11* |
|  | 2 | 866319～6545390 | 0.01515 | 866319～1245368 | 0.10606 |  |  |  |
|  | 3 | 110282972～117660608 | 0.01515 | 111661413～112321032 | 0.07576 |  |  |  |
| **2** | 1 | 74400532～85598056 | 0.01515 | 84518632～85598056 | 0.01515 | 84800898～84940398 | 0.01515 |  |
|  | 2 | 27033604～28268467 | 0.01515 | 27033604～28268467 | 0.01515 | 27462405～27547247 | 0.01515 |  |
|  | 3 | 120449971～128615577 | 0.01515 | 134368381～136437615 | 0.01515 | 135722143～136437615 | 0.01515 |  |
| **3** | 1 | 130020221～134138667 | 0.01515 | 130887502～133429441 | 0.01515 | 132235794～132256381 | 0.01515 | *DNAJC13* |
|  | 2 | 193047723～197838002 | 0.01515 | 196795720～197838002 | 0.01515 | 197273882～197423637 | 0.0303 |  |
|  | 3 | 48576483～49314960 | 0.01515 | 48576483～49314960 | 0.04545 | 48675856～48677621 | 0.04545 |  |
| **4** | 1 | 87256623～88904261 | 0.01515 | 87256623～88904261 | 0.01515 | 87473339～87674341 | 0.01515 |  |
|  | 2 | 48115264～58139506 | 0.01515 | 57189498～57524195 | 0.01515 | 57307642～57384689 | 0.01515 |  |
|  | 3 | 185606395～185718606 | 0.01515 | 185606395～185718606 | 0.01515 | 185606395～185654827 | 0.0303 | *PRIMPOL、CENPU* |
| **5** | 1 | 134574406～145108829 | 0.04545 | 134782576～136194526 | 0.01515 | 135398279～135512916 | 0.0303 |  |
|  | 2 | 54459961～54830629 | 0.01515 | 54459961～54830629 | 0.04545 | 54652636～54771380 | 0.07576 |  |
|  | 3 | 116340410～127121045 | 0.01515 | 122685801～127121045 | 0.01515 | 122685801～122726783 | 0.04545 | *CEP120* |
| **6** | 1 | 27792228～35109157 | 0.01515 | 27792228～30043779 | 0.01515 | 30030114～30041509 | 0.01515 |  |
|  | 2 | 12021539～24559029 | 0.01515 | 14819091～18387683 | 0.01515 | 15468632～15496662 | 0.0303 | *JARID2* |
|  | 3 | 110106234～112493717 | 0.01515 | 111374131～112493717 | 0.01515 | 112015373～112207258 | 0.01515 |  |
| **7** | 1 | 148937136～156840791 | 0.01515 | 148937136～150935430 | 0.01515 | 149474393～149497421 | 0.01515 |  |
|  | 2 | 102825871～130472991 | 0.01515 | 127950725～128767250 | 0.01515 | 128142587～128319982 | 0.01515 | *METTL2B、LOC101928451、RPS10P15、LINC01000、CICP14、FAM71F2* |
|  | 3 | 1484572～2649932 | 0.01515 | 1484572～2048136 | 0.13636 |  |  |  |
| **8** | 1 | 94716106～104453603 | 0.01515 | 98609039～103676605 | 0.04545 | 103220351～103238472 | 0.04545 |  |
|  | 2 | 20884737～24359279 | 0.01515 | 21959191～22069386 | 0.01515 | 22022749～22052894 | 0.01515 | *BMP1* |
|  | 3 | 42552908～52396241 | 0.04545 | 50892076～52396241 | 0.07576 |  |  |  |
| **9** | 1 | 87360106～99380914 | 0.01515 | 94538115～95570008 | 0.01515 | 95068046～95084066 | 0.04545 |  |
|  | 2 | 132410768～132662585 | 0.01515 | 132410768～132662585 | 0.01515 | 132623075～132631680 | 0.01515 |  |
|  | 3 | 34410165～36249016 | 0.01515 | 35757362～36211827 | 0.01515 | 36019814～36148722 | 0.01515 |  |
| **10** | 1 | 80813364～93031580 | 0.01515 | 103281722～103702763 | 0.04545 | 103281722～103384849 | 0.01515 | *BTRC、DPCD、POLL、MIR3158-1、MIR3158-2、FBXW4* |
|  | 2 | 70097281～70287352 | 0.01515 | 70097281～70287352 | 0.01515 | 70097281～70143371 | 0.07576 |  |
|  | 3 | 32122419～32398895 | 0.04545 | 32269363～32398895 | 0.04545 | 32321441～32345138 | 0.01515 | *KIF5B* |
| **11** | 1 | 76734957～86577952 | 0.01515 | 76782378～86111641 | 0.01515 | 76831622～76834712 | 0.01515 | *CAPN5* |
|  | 2 | 64702200～65319969 | 0.01515 | 64702200～65319969 | 0.04545 | 64799894～64807447 | 0.01515 |  |
|  | 3 | 130165697～134153518 | 0.01515 | 133853694～134153518 | 0.01515 | 134105165～134120325 | 0.0303 |  |
| **12** | 1 | 109639609～117665599 | 0.01515 | 109639609～117665599 | 0.01515 | 113796566～113874497 | 0.01515 |  |
|  | 2 | 48104746～50348359 | 0.01515 | 48104746～50348359 | 0.01515 | 48740879～48876902 | 0.01515 | *ZNF641、OR5BK1P、OR5BT1P、OR5BJ1P、OR8S21P、OR8T1P、ANP32D、C12orf54* |
|  | 3 | 69967636～79701964 | 0.01515 | 69967636～77077984 | 0.01515 | 70965902～70988287 | 0.01515 |  |
| **13** | 1 | 19601182～19943770 | 0.01515 | 19601182～19943770 | 0.01515 | 19751544～19755387 | 0.01515 |  |
|  | 2 | 96796392～99096204 | 0.25758 |  |  |  |  |  |
|  | 3 | 103082595～103346659 | 0.04545 | 103082595～103346659 | 0.10606 |  |  |  |
| **14** | 1 | 39017903～45711594 | 0.01515 | 39645863～45711594 | 0.01515 | 45128574～45711594 | 0.01515 |  |
|  | 2 | 59105188～60625999 | 0.01515 | 59950417～60625999 | 0.01515 | 60080424～60625999 | 0.0303 |  |
|  | 3 | 101302839～102973900 | 0.01515 | 101442218～102973900 | 0.07576 |  |  |  |
| **15** | 1 | 58860963～59499274 | 0.04545 | 58860963～59499274 | 0.01515 | 58920275～58982987 | 0.0303 | *ADAM10、LOC100289060、HSP90AB4P* |
|  | 2 | 25184577～31294702 | 0.01515 | 25184577～25981367 | 0.01515 | 25514778～25548205 | 0.01515 |  |
|  | 3 | 48595192～53621597 | 0.01515 | 51981291～53621597 | 0.01515 | 52628489～52667450 | 0.01515 |  |
| **16** | 1 | 688557～1085393 | 0.04545 | 688557～1085393 | 0.04545 | 716273～717057 | 0.0303 |  |
|  | 2 | 17337724～23646191 | 0.04545 | 20552075～21183310 | 0.13636 |  |  |  |
|  | 3 | 31004169～31368874 | 0.07576 |  |  |  |  |  |
| **17** | 1 | 5276934～6719367 | 0.07576 |  |  |  |  |  |
|  | 2 | 39018961～39156084 | 0.01515 | 39018961～39156084 | 0.01515 | 39123013～39135084 | 0.01515 |  |
|  | 3 | 56083934～58503732 | 0.01515 | 56083934～58503732 | 0.04545 | 56711542～56769979 | 0.0303 | *TEX14、IGBP1P2、RAD51C* |
| **18** | 1 | 61410201～61637225 | 0.04545 | 61410201～61637225 | 0.01515 | 61509075～61637225 | 0.01515 | *SERPINB2、SERPINB10、HMSD* |
|  | 2 | 76389156～77137155 | 0.04545 | 76389156～77107488 | 0.01515 | 77067000～77107488 | 0.01515 |  |
|  | 3 | 56517508～59530487 | 0.01515 | 56517508～59530487 | 0.04545 | 57836715～58089214 | 0.01515 |  |
| **19** | 1 | 36288400～41703792 | 0.01515 | 36288400～41703792 | 0.01515 | 41019878～41073354 | 0.0303 | *SPTBN4* |
|  | 2 | 49300605～52839479 | 0.01515 | 52332525～52839479 | 0.01515 | 52332525～52376448 | 0.0303 |  |
|  | 3 | 20298670～21145956 | 0.01515 | 20878430～21145956 | 0.04545 | 21125524～21145956 | 0.04545 |  |
| **20** | 1 | 61885887～62554114 | 0.01515 | 61885887～61960013 | 0.01515 | 61917241～61926723 | 0.07576 |  |
|  | 2 | 31531544～58251012 | 0.01515 | 37314182～43926536 | 0.07576 |  |  |  |
|  | 3 | 453101～2448559 | 0.04545 | 1312910～2233913 | 0.04545 | 2142755～2233913 | 0.06061 |  |
| **21** | 1 | 30257729～30505934 | 0.25758 |  |  |  |  |  |
|  | 2 | 32526841～34985754 | 0.01515 | 34668747～34985754 | 0.04545 | 34839533～34892896 | 0.0303 | *TMEM50B、RPS5P3、DNAJC28、GART、BTF3P6* |
|  | 3 | 42925376～43289997 | 0.01515 | 43236252～43289997 | 0.01515 | 43258000～43279137 | 0.01515 |  |
| **22** | 1 | 46607613～46835338 | 0.01515 | 46607613～46835338 | 0.01515 | 46627603～46652929 | 0.01515 |  |
|  | 2 | 26924456～33828097 | 0.01515 | 28122786～33828097 | 0.01515 | 29191879～29350370 | 0.01515 |  |
|  | 3 | 19156117～19867914 | 0.01515 | 19156117～19867914 | 0.01515 | 19214071～19215560 | 0.01515 |  |

Patients MIA (F1+F4+F6)

| **chr** | **rank** | **eMSS** | **p-value** | **1st eMSS refinement** | **p-value** | **2nd eMSS refinement** | **p-value** | **Gene symbol** |
| --- | --- | --- | --- | --- | --- | --- | --- | --- |
| **1** | 1 | 224517907～236209081 | 0.99751 |  |  |  |  |  |
|  | 2 | 36955700～46871986 | 0.21642 |  |  |  |  |  |
|  | 3 | 110194255～113658887 | 0.10199 |  |  |  |  |  |
| **2** | 1 | 38916906～68676008 | 0.00249 | 38916906～55662790 | 0.00249 | 47133330～47277043 | 0.00249 | *MCFD2, TTC7A* |
|  | 2 | 10912153～10959475 | 0.45025 |  |  |  |  |  |
|  | 3 | 23865260～24245659 | 0.15174 |  |  |  |  |  |
| **3** | 1 | 156742775～196054488 | 0.74876 |  |  |  |  |  |
|  | 2 | 12875443～36809360 | 0.06716 |  |  |  |  |  |
|  | 3 | 50329826～60717960 | 0.00746 | 50329826～58494546 | 0.62935 |  |  |  |
| **4** | 1 | 79300656～106861730 | 0.85821 |  |  |  |  |  |
|  | 2 | 5966618～9700910 | 0.73881 |  |  |  |  |  |
|  | 3 | 47525009～48170669 | 0.37065 |  |  |  |  |  |
| **5** | 1 | 68470527～77081608 | 0.96766 |  |  |  |  |  |
|  | 2 | 163260～10448247 | 0.21642 |  |  |  |  |  |
|  | 3 | 54652636～54847837 | 0.06965 |  |  |  |  |  |
| **6** | 1 | 27792228～29576393 | 0.99751 |  |  |  |  |  |
|  | 2 | 10931587～16290862 | 0.45522 |  |  |  |  |  |
|  | 3 | 160468383～169640529 | 0.17662 |  |  |  |  |  |
| **7** | 1 | 128141638～141797564 | 0.99751 |  |  |  |  |  |
|  | 2 | 55259763～87092185 | 0.02736 | 62672146～64490841 | 0.65423 |  |  |  |
|  | 3 | 154461112～157370688 | 0.00249 | 154461112～157370688 | 0.22637 |  |  |  |
| **8** | 1 | 6886855～12600720 | 0.86318 |  |  |  |  |  |
|  | 2 | 87494974～124553222 | 0.36567 |  |  |  |  |  |
|  | 3 | 27598172～28588904 | 0.301 |  |  |  |  |  |
| **9** | 1 | 132402908～139621120 | 0.99751 |  |  |  |  |  |
|  | 2 | 36218018～98112157 | 0.00249 | 37426804～98112157 | 0.93284 |  |  |  |
|  | 3 | 4793254～11012115 | 0.02736 | 6984620～7174430 | 0.71891 |  |  |  |
| **10** | 1 | 101558746～104378750 | 0.77363 |  |  |  |  |  |
|  | 2 | 32404788～45877899 | 0.40547 |  |  |  |  |  |
|  | 3 | 89705429～91200711 | 0.4801 |  |  |  |  |  |
| **11** | 1 | 5842628～18267027 | 0.91791 |  |  |  |  |  |
|  | 2 | 58207204～63138482 | 0.72886 |  |  |  |  |  |
|  | 3 | 71529252～75430950 | 0.301 |  |  |  |  |  |
| **12** | 1 | 70147464～123345736 | 0.61443 |  |  |  |  |  |
|  | 2 | 5961048～8289300 | 0.23134 |  |  |  |  |  |
|  | 3 | 53002956～56232176 | 0.03731 | 53002956～53451952 | 0.38557 |  |  |  |
| **13** | 1 | 96484751～99447005 | 0.98259 |  |  |  |  |  |
|  | 2 | 37316072～39544663 | 0.17164 |  |  |  |  |  |
|  | 3 | 79483726～81194086 | 0.05224 |  |  |  |  |  |
| **14** | 1 | 23079730～39856043 | 0.94776 |  |  |  |  |  |
|  | 2 | 60213375～61038944 | 0.17662 |  |  |  |  |  |
|  | 3 | 91666018～94594768 | 0.18159 |  |  |  |  |  |
| **15** | 1 | 43632549～45706739 | 0.71891 |  |  |  |  |  |
|  | 2 | 90768156～91177939 | 0.35572 |  |  |  |  |  |
|  | 3 | 38233869～40226495 | 0.68408 |  |  |  |  |  |
| **16** | 1 | 23713395～28898793 | 0.89801 |  |  |  |  |  |
|  | 2 | 2375961～4414987 | 0.44527 |  |  |  |  |  |
|  | 3 | 81157324～81510155 | 0.22139 |  |  |  |  |  |
| **17** | 1 | 80008676～80602050 | 0.99751 |  |  |  |  |  |
|  | 2 | 71223108～73597544 | 0.17662 |  |  |  |  |  |
|  | 3 | 34951327～39593768 | 0.06716 |  |  |  |  |  |
| **18** | 1 | 29126670～33647254 | 0.99751 |  |  |  |  |  |
|  | 2 | 56390553～61170500 | 0.17662 |  |  |  |  |  |
|  | 3 | 1278665～2909831 | 0.03234 | 2707619～2909831 | 0.40547 |  |  |  |
| **19** | 1 | 35051660～36276086 | 0.73383 |  |  |  |  |  |
|  | 2 | 44975752～52869534 | 0.3607 |  |  |  |  |  |
|  | 3 | 15890625～16254694 | 0.21144 |  |  |  |  |  |
| **20** | 1 | 25479058～31446645 | 0.301 |  |  |  |  |  |
|  | 2 | 44170712～55071620 | 0.02736 | 50705584～55071620 | 0.25124 |  |  |  |
|  | 3 | 2779257～10330136 | 0.04229 | 8719777～10330136 | 0.10199 |  |  |  |
| **21** | 1 | 45970812～47417303 | 0.78856 |  |  |  |  |  |
|  | 2 | 41150853～43547788 | 0.27612 |  |  |  |  |  |
|  | 3 | 26734301～26979628 | 0.01741 | 26734301～26979628 | 0.59204 |  |  |  |
| **22** | 1 | 23104867～30384572 | 0.96269 |  |  |  |  |  |
|  | 2 | 41753569～42166486 | 0.12189 |  |  |  |  |  |
|  | 3 | 17600148～19754091 | 0.04726 | 17600148～19754091 | 0.59453 |  |  |  |

Patients F1.

| **chr** | **rank** | **eMSS** | **p-value** | **1st eMSS refinement** | **p-value** | **2nd eMSS refinement** | **p-value** | **Gene symbol** |
| --- | --- | --- | --- | --- | --- | --- | --- | --- |
| **1** | 1 | 11837299～20664656 | 0.01515 | 19149639～20107018 | 0.04545 | 19600323～19600395 | 0.06061 |  |
|  | 2 | 224762167～226549498 | 0.01515 | 224762167～226019371 | 0.01515 | 225230904～225410051 | 0.01515 |  |
|  | 3 | 50871649～55253594 | 0.01515 | 50871649～53553657 | 0.01515 | 53422823～53493822 | 0.01515 |  |
| **2** | 1 | 132594079～138771760 | 0.01515 | 132796480～136873084 | 0.01515 | 135745722～136422784 | 0.01515 |  |
|  | 2 | 45205547～71635931 | 0.01515 | 45205547～47961712 | 0.01515 | 47251634～47256618 | 0.0303 | *TTC7A* |
|  | 3 | 162175290～170917544 | 0.01515 | 170145426～170917544 | 0.01515 | 170606724～170644364 | 0.0303 | *PHOSPHO2-KLHL23、KLHL23、LOC171417、PTCHD3P2* |
| **3** | 1 | 156711204～182554048 | 0.01515 | 156711204～157160061 | 0.01515 | 156830569～157081604 | 0.01515 |  |
|  | 2 | 54798258～58516383 | 0.01515 | 58089877～58516383 | 0.01515 | 58385194～58398447 | 0.0303 |  |
|  | 3 | 12914900～15687206 | 0.01515 | 13670837～15687206 | 0.01515 | 15371819～15456949 | 0.06061 |  |
| **4** | 1 | 69687987～71471905 | 0.04545 | 69687987～71471905 | 0.04545 | 71389865～71420264 | 0.01515 | *AMTN* |
|  | 2 | 8961475～9711314 | 0.04545 | 8961475～9711314 | 0.04545 | 9384846～9387204 | 0.07576 |  |
|  | 3 | 83196452～103534425 | 0.04545 | 83196452～83774687 | 0.28788 |  |  |  |
| **5** | 1 | 58298～922545 | 0.10606 |  |  |  |  |  |
|  | 2 | 102891695～110084154 | 0.01515 | 108014484～110084154 | 0.01515 | 108014484～108233158 | 0.01515 |  |
|  | 3 | 123399421～130846190 | 0.01515 | 126525350～127638759 | 0.01515 | 126781446～126791229 | 0.06061 |  |
| **6** | 1 | 29940488～30042668 | 0.04545 | 29940488～30042668 | 0.01515 | 29963065～29969973 | 0.01515 |  |
|  | 2 | 55765071～65767393 | 0.01515 | 55765071～65767393 | 0.01515 | 56873069～56917538 | 0.01515 | *BEND6、MRPL30P1、KIAA1586* |
|  | 3 | 110883876～112029331 | 0.01515 | 110883876～111346701 | 0.01515 | 111245074～111318588 | 0.01515 | *LOC442244、GTF3C6、RPF2* |
| **7** | 1 | 135048497～141805522 | 0.01515 | 138883381～141797564 | 0.01515 | 141512088～141537563 | 0.0303 | *PRSS37* |
|  | 2 | 84647477～92938116 | 0.01515 | 84647477～92938116 | 0.01515 | 87032613～87053150 | 0.0303 |  |
|  | 3 | 55629723～57250003 | 0.01515 | 55808713～57250003 | 0.01515 | 56136260～56149939 | 0.0303 |  |
| **8** | 1 | 110396078～126096274 | 0.01515 | 126016021～126096274 | 0.04545 | 126085363～126095503 | 0.01515 |  |
|  | 2 | 41530482～84325432 | 0.01515 | 41530482～43528072 | 0.04545 | 42552908～42587659 | 0.04545 |  |
|  | 3 | 25246481～26869815 | 0.04545 | 26505518～26869815 | 0.04545 | 26716472～26716715 | 0.04545 | *ADRA1A* |
| **9** | 1 | 98678164～116450116 | 0.01515 | 98678164～116450116 | 0.01515 | 99314012～99614104 | 0.01515 |  |
|  | 2 | 1057318～33113970 | 0.01515 | 1057318～5929167 | 0.01515 | 3270683～3452301 | 0.0303 |  |
|  | 3 | 136870242～139265230 | 0.01515 | 138646881～138838305 | 0.10606 |  |  |  |
| **10** | 1 | 102104521～102586723 | 0.22727 |  |  |  |  |  |
|  | 2 | 76729632～84922481 | 0.01515 | 94824488～95185696 | 0.01515 | 95164040～95185696 | 0.01515 |  |
|  | 3 | 34666864～38357629 | 0.01515 | 35324076～38357629 | 0.04545 | 38108176～38172608 | 0.01515 |  |
| **11** | 1 | 71381285～72091228 | 0.01515 | 71381285～72091228 | 0.01515 | 71529252～71630226 | 0.01515 |  |
|  | 2 | 407708～1032818 | 0.01515 | 407708～1032818 | 0.01515 | 486855～489829 | 0.0303 |  |
|  | 3 | 113318408～117670752 | 0.01515 | 113679119～114653591 | 0.01515 | 114393314～114442265 | 0.0303 | *NXPE1、LOC101928962、NXPE4* |
| **12** | 1 | 40825169～41421829 | 0.01515 | 40825169～41421829 | 0.01515 | 40905210～40916874 | 0.0303 | *MUC19* |
|  | 2 | 121097622～126932624 | 0.01515 | 121097622～126932624 | 0.01515 | 122692820～123024476 | 0.01515 |  |
|  | 3 | 53111528～53433788 | 0.01515 | 53111528～53433788 | 0.01515 | 53227803～53242641 | 0.01515 |  |
| **13** | 1 | 96484751～102030837 | 0.07576 |  |  |  |  |  |
|  | 2 | 42044508～42769267 | 0.01515 | 42044508～42769267 | 0.01515 | 42748136～42761457 | 0.01515 |  |
|  | 3 | 61355223～76180042 | 0.01515 | 69559248～76180042 | 0.01515 | 73358047～73574451 | 0.01515 | *PIBF1、RNU6-79P* |
| **14** | 1 | 60426771～61068725 | 0.01515 | 60426771～61068725 | 0.01515 | 60426771～60625999 | 0.01515 |  |
|  | 2 | 23855849～24731622 | 0.01515 | 23855849～24731622 | 0.01515 | 24647957～24657226 | 0.01515 | *REC8、IPO4* |
|  | 3 | 38321611～45759378 | 0.04545 | 42716716～45759378 | 0.04545 | 44976241～45335275 | 0.01515 |  |
| **15** | 1 | 42437711～45848153 | 0.04545 | 42437711～45848153 | 0.04545 | 45391566～45399467 | 0.09091 |  |
|  | 2 | 65693397～68266880 | 0.01515 | 65693397～68266880 | 0.01515 | 66821174～66857791 | 0.0303 | *ZWILCH、LCTL* |
|  | 3 | 82512429～84281176 | 0.01515 | 82512429～84281176 | 0.04545 | 83240293～83296131 | 0.01515 |  |
| **16** | 1 | 58703966～88132812 | 0.01515 | 66762747～68399102 | 0.01515 | 67221050～67324827 | 0.01515 |  |
|  | 2 | 11056618～15853596 | 0.34848 |  |  |  |  |  |
|  | 3 | 2345388～3794986 | 0.25758 |  |  |  |  |  |
| **17** | 1 | 43344073～44967530 | 0.01515 | 43344073～44828931 | 0.01515 | 43516738～43555253 | 0.04545 | *PLEKHM1、MIR4315-1* |
|  | 2 | 39296016～39535388 | 0.01515 | 39296016～39535388 | 0.04545 | 39296016～39341266 | 0.01515 |  |
|  | 3 | 73816026～73944596 | 0.01515 | 73816026～73944596 | 0.07576 |  |  |  |
| **18** | 1 | 22030585～30846895 | 0.19697 |  |  |  |  |  |
|  | 2 | 11861282～12014220 | 0.07576 |  |  |  |  |  |
|  | 3 | 15005736～20956908 | 0.04545 | 15005736～20956908 | 0.13636 |  |  |  |
| **19** | 1 | 55085120～55715090 | 0.01515 | 55085120～55505557 | 0.01515 | 55143491～55147869 | 0.01515 |  |
|  | 2 | 22715987～23329860 | 0.01515 | 22715987～23158640 | 0.01515 | 22793083～22942061 | 0.01515 |  |
|  | 3 | 43343882～53577214 | 0.01515 | 53321445～53540130 | 0.04545 | 53452965～53474109 | 0.01515 |  |
| **20** | 1 | 33763951～40714218 | 0.04545 | 33763951～40714218 | 0.04545 | 34063095～34135374 | 0.06061 |  |
|  | 2 | 25197982～31062176 | 0.01515 | 25197982～31062176 | 0.01515 | 25249734～25263895 | 0.01515 |  |
|  | 3 | 50769549～52775774 | 0.16667 |  |  |  |  |  |
| **21** | 1 | 45175958～47570654 | 0.01515 | 45175958～45560236 | 0.01515 | 45337152～45506819 | 0.01515 |  |
|  | 2 | 24473661～27141292 | 0.01515 | 24473661～27141292 | 0.04545 | 27009324～27078428 | 0.01515 |  |
|  | 3 | 41898497～42842591 | 0.01515 | 42622786～42842591 | 0.01515 | 42694667～42735872 | 0.01515 |  |
| **22** | 1 | 31583007～32334021 | 0.04545 | 31583007～32334021 | 0.01515 | 32013265～32113269 | 0.01515 |  |
|  | 2 | 17978311～20100158 | 0.01515 | 19119938～19423250 | 0.01515 | 19371052～19384589 | 0.0303 |  |
|  | 3 | 29881884～30648502 | 0.01515 | 29881884～30648502 | 0.01515 | 29881884～29908072 | 0.01515 | *NEFH、THOC5* |

Patients F4.

| **chr** | **rank** | **eMSS** | **p-value** | **1st eMSS refinement** | **p-value** | **2nd eMSS refinement** | **p-value** | **Gene symbol** |
| --- | --- | --- | --- | --- | --- | --- | --- | --- |
| **1** | 1 | 158066834～160465800 | 0.10606 |  |  |  |  |  |
|  | 2 | 202287813～231376806 | 0.01515 | 228103428～228480964 | 0.07576 |  |  |  |
|  | 3 | 19243589～20141148 | 0.01515 | 19243589～20141148 | 0.07576 |  |  |  |
| **2** | 1 | 39108773～55528514 | 0.01515 | 45879610～55528514 | 0.01515 | 47133330～47273668 | 0.01515 | *MCFD2, TTC7A* |
|  | 2 | 132796480～137852891 | 0.01515 | 132796480～137852891 | 0.01515 | 135745129～136467119 | 0.01515 |  |
|  | 3 | 159912703～166027187 | 0.01515 | 159912703～160664907 | 0.01515 | 160005384～160084263 | 0.01515 |  |
| **3** | 1 | 183624447～185882922 | 0.04545 | 183624447～185882922 | 0.01515 | 184295316～184297942 | 0.04545 |  |
|  | 2 | 38409600～42931965 | 0.01515 | 41607701～42931965 | 0.01515 | 42917047～42931965 | 0.01515 |  |
|  | 3 | 124352573～126749017 | 0.01515 | 126270757～126749017 | 0.19697 |  |  |  |
| **4** | 1 | 87256623～89319296 | 0.01515 | 87256623～88814989 | 0.01515 | 87684031～87705862 | 0.09091 |  |
|  | 2 | 1606390～3943173 | 0.01515 | 3039150～3943173 | 0.07576 |  |  |  |
|  | 3 | 76846755～77134786 | 0.01515 | 76846755～77134786 | 0.01515 | 76847215～76857395 | 0.0303 | *NAAA* |
| **5** | 1 | 54557438～56178111 | 0.10606 |  |  |  |  |  |
|  | 2 | 784427～1007628 | 0.01515 | 784427～1007628 | 0.01515 | 817174～1007628 | 0.01515 |  |
|  | 3 | 101816327～108206968 | 0.01515 | 101816327～102537379 | 0.01515 | 102325878～102360747 | 0.04545 | *PAM* |
| **6** | 1 | 25701691～30862440 | 0.01515 | 27792228～30710135 | 0.01515 | 30668488～30679333 | 0.01515 |  |
|  | 2 | 121526465～121655392 | 0.01515 | 121577370～121655392 | 0.01515 | 121577370～121620474 | 0.01515 |  |
|  | 3 | 13978275～17531964 | 0.01515 | 14244517～17531964 | 0.01515 | 16145325～16279189 | 0.01515 |  |
| **7** | 1 | 53302338～73020337 | 0.01515 | 71571436～73020337 | 0.07576 |  |  |  |
|  | 2 | 98779740～100606141 | 0.01515 | 98779740～99361466 | 0.01515 | 99217424～99293358 | 0.01515 |  |
|  | 3 | 38796748～44252018 | 0.01515 | 43548832～44240664 | 0.01515 | 43975601～44053953 | 0.0303 | *UBE2D4、POLR2J4、SPDYE1* |
| **8** | 1 | 100133706～105263751 | 0.10606 |  |  |  |  |  |
|  | 2 | 79635864～82670771 | 0.01515 | 79635864～82670771 | 0.01515 | 82355621～82437457 | 0.01515 |  |
|  | 3 | 135505969～142481511 | 0.01515 | 135505969～139672602 | 0.07576 |  |  |  |
| **9** | 1 | 91628546～95420983 | 0.01515 | 91628546～95420983 | 0.01515 | 94768542～94842048 | 0.0303 | *RPL21P82、HSPE1P22、SPTLC1* |
|  | 2 | 132412706～134398534 | 0.01515 | 132569553～132636920 | 0.09091 |  |  |  |
|  | 3 | 38453263～72939125 | 0.01515 | 44097407～72939125 | 0.01515 | 72785653～72785845 | 0.0303 |  |
| **10** | 1 | 75863750～81932680 | 0.01515 | 76861680～81932680 | 0.01515 | 81679949～81680538 | 0.10606 |  |
|  | 2 | 43132232～50014328 | 0.01515 | 43695049～50014328 | 0.01515 | 49987020～49998824 | 0.01515 |  |
|  | 3 | 102783955～104140350 | 0.01515 | 102783955～104140350 | 0.07576 |  |  |  |
| **11** | 1 | 49013741～56431075 | 0.01515 | 49070328～56431075 | 0.01515 | 55734972～55736066 | 0.07576 |  |
|  | 2 | 5566365～6816875 | 0.04545 | 6622857～6737979 | 0.01515 | 6629530～6637504 | 0.01515 |  |
|  | 3 | 116078953～117109155 | 0.01515 | 116078953～117109155 | 0.01515 | 116701535～116707684 | 0.01515 |  |
| **12** | 1 | 48374513～122189933 | 0.01515 | 48407987～50347688 | 0.01515 | 48554258～48597096 | 0.06061 |  |
|  | 2 | 8090839～8914709 | 0.13636 |  |  |  |  |  |
|  | 3 | 302208～2368396 | 0.25758 |  |  |  |  |  |
| **13** | 1 | 27829310～28387130 | 0.16667 |  |  |  |  |  |
|  | 2 | 111176393～111563052 | 0.01515 | 111176393～111329119 | 0.01515 | 111274915～111278310 | 0.01515 | *CARKD* |
|  | 3 | 53297962～56038253 | 0.01515 | 53297962～56038253 | 0.01515 | 53941751～53983466 | 0.06061 |  |
| **14** | 1 | 94464432～102909991 | 0.04545 | 101447517～102909991 | 0.01515 | 102695693～102698599 | 0.01515 |  |
|  | 2 | 65584223～75377008 | 0.01515 | 65681808～75377008 | 0.01515 | 65701285～65747837 | 0.01515 | *LOC100420096、RPL21P7、RPL36AP2、PTBP1P* |
|  | 3 | 23532865～45696937 | 0.01515 | 23532865～24534781 | 0.01515 | 24408954～24423007 | 0.07576 |  |
| **15** | 1 | 78012713～102004368 | 0.01515 | 78012713～78454795 | 0.04545 | 78189370～78190308 | 0.04545 |  |
|  | 2 | 38750987～44856873 | 0.01515 | 41827655～43478113 | 0.07576 |  |  |  |
|  | 3 | 71522857～74003533 | 0.01515 | 72462165～74003533 | 0.01515 | 73848808～74003533 | 0.12121 |  |
| **16** | 1 | 19505852～48227862 | 0.07576 |  |  |  |  |  |
|  | 2 | 74876991～75783465 | 0.07576 |  |  |  |  |  |
|  | 3 | 57059427～68358788 | 0.01515 | 57059427～57101552 | 0.13636 |  |  |  |
| **17** | 1 | 76800182～80899349 | 0.10606 |  |  |  |  |  |
|  | 2 | 16251859～17326363 | 0.04545 | 16251859～17326363 | 0.01515 | 17131018～17168427 | 0.01515 |  |
|  | 3 | 34067892～37253064 | 0.01515 | 34067892～37253064 | 0.07576 |  |  |  |
| **18** | 1 | 30977255～33647254 | 0.07576 |  |  |  |  |  |
|  | 2 | 6948614～7039943 | 0.01515 | 6948614～7039943 | 0.01515 | 6951060～6978345 | 0.01515 | *LAMA1、LOC101927188* |
|  | 3 | 74778515～77170542 | 0.01515 | 74778515～77170542 | 0.01515 | 76754466～76812962 | 0.01515 | *SALL3* |
| **19** | 1 | 22551462～39234511 | 0.01515 | 22551462～39234511 | 0.01515 | 22793083～22868799 | 0.01515 |  |
|  | 2 | 287970～6459677 | 0.01515 | 5957683～6459677 | 0.07576 |  |  |  |
|  | 3 | 11242044～11834270 | 0.01515 | 11327626～11834270 | 0.01515 | 11327626～11348960 | 0.01515 |  |
| **20** | 1 | 33982435～56139365 | 0.01515 | 33982435～34596371 | 0.01515 | 34328848～34571873 | 0.01515 |  |
|  | 2 | 5513921～13074235 | 0.01515 | 5513921～9370861 | 0.04545 | 8770932～8812709 | 0.01515 |  |
|  | 3 | 31622083～31656938 | 0.09091 |  |  |  |  |  |
| **21** | 1 | 47642016～48063256 | 0.01515 | 47821726～47862287 | 0.01515 | 47821726～47847757 | 0.01515 |  |
|  | 2 | 43315057～45503794 | 0.01515 | 43315057～45503794 | 0.01515 | 45138420～45173705 | 0.04545 |  |
|  | 3 | 30871821～33876041 | 0.01515 | 32435489～33876041 | 0.04545 | 32435489～32513323 | 0.0303 |  |
| **22** | 1 | 17680519～21008167 | 0.01515 | 19122498～20129106 | 0.01515 | 19122498～19158756 | 0.01515 |  |
|  | 2 | 31838085～35560444 | 0.04545 | 32110163～32554996 | 0.04545 | 32482321～32506143 | 0.07576 |  |
|  | 3 | 42472237～43023980 | 0.04545 | 42472237～43023980 | 0.10606 |  |  |  |

Patients F6.

| **chr** | **rank** | **eMSS** | **p-value** | **1st eMSS refinement** | **p-value** | **2nd eMSS refinement** | **p-value** | **Gene symbol** |
| --- | --- | --- | --- | --- | --- | --- | --- | --- |
| **1** | 1 | 224621368～235806045 | 0.01515 | 224621368～226554951 | 0.07576 |  |  |  |
|  | 2 | 240991571～248685719 | 0.01515 | 248138642～248551883 | 0.04545 | 248138848～248512498 | 0.01515 | *OR2L13、OR2L9P、OR2L1P、OR2L6P、OR2L5、OR2L2、OR2L3、OR2T32P、OR2M1P、OR2M5、LOC100216488、OR2M2、OR2M3、OR2M4、OR2T33、OR2T12、OR2M7、OR14C36* |
|  | 3 | 205014930～208212224 | 0.01515 | 205014930～206603535 | 0.01515 | 205031449～205034723 | 0.01515 | *CNTN2* |
| **2** | 1 | 45171842～85059227 | 0.01515 | 45171842～52303524 | 0.04545 | 47133330～47273668 | 0.01515 | *MCFD2, TTC7A* |
|  | 2 | 239056867～242925684 | 0.01515 | 241828034～242121959 | 0.01515 | 242021742～242046785 | 0.06061 |  |
|  | 3 | 179118607～184125985 | 0.01515 | 179600563～184125985 | 0.04545 | 179600563～179613651 | 0.0303 |  |
| **3** | 1 | 123988039～178936227 | 0.01515 | 125029761～125831799 | 0.04545 | 125301711～125422862 | 0.06061 |  |
|  | 2 | 11950507～38307656 | 0.01515 | 33626229～38307656 | 0.04545 | 33863705～33958926 | 0.01515 |  |
|  | 3 | 50416501～52812826 | 0.04545 | 50416501～51813018 | 0.16667 |  |  |  |
| **4** | 1 | 110896050～124774867 | 0.01515 | 113554385～124774867 | 0.01515 | 119736598～119754918 | 0.0303 |  |
|  | 2 | 88296092～106890187 | 0.04545 | 106082120～106890187 | 0.10606 | 106848703～106861557 | 0.01515 |  |
|  | 3 | 69853310～70116534 | 0.07576 |  |  |  |  |  |
| **5** | 1 | 95018382～103141266 | 0.01515 | 95018382～103141266 | 0.01515 | 102444089～102491502 | 0.04545 | *GIN1、PPIP5K2* |
|  | 2 | 148675225～150666962 | 0.01515 | 149678296～150647156 | 0.01515 | 150076556～150174810 | 0.07576 |  |
|  | 3 | 54993829～56180480 | 0.01515 | 54993829～56180480 | 0.01515 | 55082474～55168132 | 0.01515 |  |
| **6** | 1 | 32727004～34949607 | 0.01515 | 32803840～34026938 | 0.01515 | 33048542～33074908 | 0.01515 | *HLA-DPA1、HLA-DPB1、HLA-DPA2、COL11A2P1* |
|  | 2 | 29274486～30041559 | 0.01515 | 29365423～30041559 | 0.01515 | 29394427～29455811 | 0.01515 |  |
|  | 3 | 149813362～151789282 | 0.01515 | 149813362～151789282 | 0.01515 | 150263186～150298543 | 0.04545 |  |
| **7** | 1 | 62615322～65444288 | 0.01515 | 62615322～63981425 | 0.01515 | 62694258～62941320 | 0.06061 |  |
|  | 2 | 142495308～143002246 | 0.01515 | 142495308～143002246 | 0.01515 | 142541619～142565776 | 0.01515 |  |
|  | 3 | 131815343～137570321 | 0.01515 | 131815343～133906669 | 0.07576 |  |  |  |
| **8** | 1 | 100298198～104225536 | 0.01515 | 100298198～103373675 | 0.01515 | 103291591～103316572 | 0.01515 |  |
|  | 2 | 79635969～82670771 | 0.01515 | 79635969～82670771 | 0.01515 | 82355621～82392918 | 0.04545 |  |
|  | 3 | 39644691～42829176 | 0.01515 | 40554994～42829176 | 0.07576 |  |  |  |
| **9** | 1 | 136137646～136291469 | 0.04545 | 136213056～136291469 | 0.01515 | 136234124～136267149 | 0.01515 |  |
|  | 2 | 140392494～140970097 | 0.01515 | 140605355～140970097 | 0.01515 | 140605355～140970097 | 0.01515 |  |
|  | 3 | 85914243～94919786 | 0.01515 | 91628546～94919786 | 0.07576 |  |  |  |
| **10** | 1 | 64426056～70266546 | 0.19697 |  |  |  |  |  |
|  | 2 | 81886064～82842535 | 0.01515 | 81901722～82842535 | 0.01515 | 81926702～81932680 | 0.09091 |  |
|  | 3 | 45953767～46280568 | 0.01515 | 45953767～46280568 | 0.04545 | 45953767～46213174 | 0.04545 |  |
| **11** | 1 | 197557～654091 | 0.22727 |  |  |  |  |  |
|  | 2 | 3128380～3469729 | 0.01515 | 3128380～3469729 | 0.01515 | 3382069～3431920 | 0.01515 | *ZNF195、LOC650368、OR7E12P、FAM86GP* |
|  | 3 | 77884782～85193027 | 0.01515 | 77884782～85193027 | 0.01515 | 77960888～78128797 | 0.22727 |  |
| **12** | 1 | 40632099～40827891 | 0.01515 | 40715013～40827891 | 0.01515 | 40802150～40815261 | 0.04545 |  |
|  | 2 | 96694162～108154199 | 0.01515 | 120270528～120884291 | 0.04545 | 120527599～120546145 | 0.0303 |  |
|  | 3 | 6560573～7283134 | 0.01515 | 6560573～7648080 | 0.01515 | 6948692～7053149 | 0.01515 |  |
| **13** | 1 | 25021358～25283744 | 0.10606 |  |  |  |  |  |
|  | 2 | 37014366～39100848 | 0.07576 |  |  |  |  |  |
|  | 3 | 75903368～77531115 | 0.19697 |  |  |  |  |  |
| **14** | 1 | 64564527～74567952 | 0.01515 | 70489680～74567952 | 0.01515 | 73409683～73425315 | 0.01515 |  |
|  | 2 | 92343764～104251276 | 0.01515 | 93617125～94156321 | 0.01515 | 93685221～93762162 | 0.01515 | *UBR7、RPL18AP1、BTBD7、RPL36AP4* |
|  | 3 | 24905533～31647439 | 0.01515 | 24905533～25736104 | 0.01515 | 24905548～24906534 | 0.07576 |  |
| **15** | 1 | 36989469～40752134 | 0.10606 |  |  |  |  |  |
|  | 2 | 42211486～42386630 | 0.01515 | 42211486～42386630 | 0.07576 |  |  |  |
|  | 3 | 73319687～74494652 | 0.01515 | 73319687～74494652 | 0.01515 | 73995431～74003533 | 0.07576 |  |
| **16** | 1 | 460543～1827522 | 0.01515 | 613168～1826023 | 0.01515 | 1660274～1712386 | 0.01515 |  |
|  | 2 | 56602671～68012694 | 0.04545 | 66420885～68012694 | 0.10606 |  |  |  |
|  | 3 | 88778133～88902484 | 0.01515 | 88787704～88902484 | 0.10606 |  |  |  |
| **17** | 1 | 33998802～37369416 | 0.04545 | 33998802～37369416 | 0.01515 | 36243668～36519221 | 0.01515 |  |
|  | 2 | 79207061～80183924 | 0.01515 | 79596811～79835930 | 0.01515 | 79769466～79805134 | 0.0303 | *GCGR、FAM195B、PPP1R27、P4HB* |
|  | 3 | 560604～3417253 | 0.01515 | 560604～3417253 | 0.01515 | 1424948～1442349 | 0.01515 |  |
| **18** | 1 | 55399268～65498790 | 0.04545 | 56279025～57014713 | 0.19697 |  |  |  |
|  | 2 | 645176～2795852 | 0.01515 | 645176～2795852 | 0.01515 | 674535～683116 | 0.04545 | *ENOSF1* |
|  | 3 | 22102471～33767749 | 0.01515 | 28660500～28728411 | 0.01515 | 28660500～28728281 | 0.01515 | *DSC2、LOC101927698、DSC1* |
| **19** | 1 | 51244631～52887282 | 0.01515 | 52248990～52887282 | 0.01515 | 52468998～52504920 | 0.01515 |  |
|  | 2 | 16594967～17113147 | 0.01515 | 16594967～17113147 | 0.01515 | 16613733～16630613 | 0.01515 |  |
|  | 3 | 42799049～44153255 | 0.01515 | 42799049～43773572 | 0.01515 | 43137202～43439528 | 0.01515 | *LIPE-AS1、CEACAMP1、CEACAMP5、RPS10P28、PSG3、PSG8、CEACAMP6 、LOC100289650、PSG10P、PSG1、PSG6、PSG7* |
| **20** | 1 | 25197982～31097877 | 0.01515 | 25197982～31097877 | 0.01515 | 25529139～25843344 | 0.01515 |  |
|  | 2 | 43929473～46044715 | 0.01515 | 43929473～46044715 | 0.01515 | 44663475～44672681 | 0.01515 |  |
|  | 3 | 8719777～11602003 | 0.10606 |  |  |  |  |  |
| **21** | 1 | 33280563～34115455 | 0.01515 | 33280563～34115455 | 0.01515 | 33694224～33719400 | 0.0303 | *URB1* |
|  | 2 | 14721911～15596683 | 0.01515 | 14760203～15306642 | 0.07576 |  |  |  |
|  | 3 | 43221483～43372219 | 0.04545 | 43221483～43372219 | 0.04545 | 43338400～43338948 | 0.06061 |  |
| **22** | 1 | 41615376～44395451 | 0.04545 | 41615376～44395451 | 0.04545 | 41745355～41753059 | 0.0303 | *ZC3H7B* |
|  | 2 | 23712647～29271088 | 0.01515 | 27038708～29271088 | 0.22727 |  |  |  |
|  | 3 | 38877106～39224123 | 0.01515 | 38877106～39224123 | 0.01515 | 39095698～39110124 | 0.09091 |  |
