# Supplementary material for: Maximal Segmental Score Method for Localizing Recessive Disease Variants Based on Sequence Data
Source: Front Genet. 2020 Jun 12;11:555. doi: 10.3389/fgene.2020.00555 (PMC7325894; doi:10.3389/fgene.2020.00555)
Supplement: Supplementary file 1 [file Presentation_1.zip › Figure S2.DOCX]

Supplementary Table S2

All shortlisted candidate regions in each of the two patients (III-5 and III-15) used in our eMSS calculations

Patients. III-5

| **chr** | **rank** | **eMSS** | **p-value** | **1st eMSS refinement** | **p-value** | **2nd eMSS refinement** | **p-value** | **Gene symbol** |
| --- | --- | --- | --- | --- | --- | --- | --- | --- |
| **1** | 1 | 998395～28921199 | 0.01515 | 10725498～15710411 | 0.01515 | 11141187～11273418 | 0.0303 | *EXOSC10、LOC101929136、MTOR、MTOR-AS1、ANGPTL7* |
|  | 2 | 229790048～235506642 | 0.01515 | 232650867～235506642 | 0.01515 | 233489529～233767059 | 0.01515 | *KIAA1804、KCNK1、MIR4427* |
|  | 3 | 83580632～85040069 | 0.01515 | 83580632～85040069 | 0.01515 | 84701251～84831134 | 0.0303 |  |
| **2** | 1 | 173292709～180610351 | 0.01515 | 179363807～180610351 | 0.01515 | 179413110～179428373 | 0.01515 |  |
|  | 2 | 74345780～99485676 | 0.01515 | 74345780～75094746 | 0.01515 | 74448672～74710491 | 0.01515 |  |
|  | 3 | 216908679～219114671 | 0.01515 | 216908679～219900068 | 0.01515 | 218712918～218770984 | 0.01515 |  |
| **3** | 1 | 10967739～38163900 | 0.01515 | 10967739～38163900 | 0.01515 | 15194949～15216703 | 0.06061 |  |
|  | 2 | 56599258～149374873 | 0.01515 | 128890350～135759669 | 0.01515 | 130129322～130134563 | 0.01515 |  |
|  | 3 | 196554020～197765432 | 0.07576 |  |  |  |  |  |
| **4** | 1 | 69796838～71509431 | 0.10606 |  |  |  |  |  |
|  | 2 | 3040279～5721021 | 0.01515 | 3040279～4322624 | 0.01515 | 3318413～3465073 | 0.01515 |  |
|  | 3 | 146046138～155508627 | 0.01515 | 146046138～152609826 | 0.04545 | 151641186～151773593 | 0.01515 |  |
| **5** | 1 | 35962984～43246057 | 0.01515 | 35962984～39377418 | 0.01515 | 38823254～38997045 | 0.01515 |  |
|  | 2 | 122950911～128326177 | 0.04545 | 122950911～126993282 | 0.10606 |  |  |  |
|  | 3 | 149776232～150282784 | 0.01515 | 149776232～150282784 | 0.01515 | 149921251～149936578 | 0.01515 |  |
| **6** | 1 | 25849620～30312881 | 0.01515 | 25849620～29342236 | 0.01515 | 28239932～28500235 | 0.01515 | *ZSCAN26、PGBD1、ZSCAN31、ZKSCAN3、ZSCAN12、ZNF968P、ZSCAN23、COX11P1、LOC101928950、OR2E1P、TRNAT16、TRNAL47P、TRNAL12、TRMEP1、TRNAT5、GPX6、GPX5* |
|  | 2 | 159205668～159660879 | 0.01515 | 159205668～159660879 | 0.01515 | 159653635～159659523 | 0.01515 |  |
|  | 3 | 2990619～3290726 | 0.01515 | 2990619～3290726 | 0.04545 | 3118947～3152822 | 0.06061 |  |
| **7** | 1 | 128415195～128767506 | 0.01515 | 128415195～128767506 | 0.01515 | 128487866～128532980 | 0.01515 |  |
|  | 2 | 98850491～100464053 | 0.01515 | 98850491～100464053 | 0.01515 | 99217424～99234899 | 0.09091 |  |
|  | 3 | 40899967～50344474 | 0.01515 | 40899967～44580876 | 0.04545 | 44442296～44580876 | 0.01515 | *NUDCD3、RPL32P18、RPL36AP27、NPC1L1* |
| **8** | 1 | 64321899～133906211 | 0.01515 | 96070181～120940652 | 0.01515 | 110516527～110657355 | 0.01515 |  |
|  | 2 | 1806229～9639316 | 0.01515 | 8887542～9639316 | 0.07576 |  |  |  |
|  | 3 | 17541999～18387267 | 0.01515 | 17541999～18387267 | 0.13636 |  |  |  |
| **9** | 1 | 91661746～110859314 | 0.01515 | 94518328～99372020 | 0.01515 | 95070095～95088255 | 0.06061 |  |
|  | 2 | 17796948～35826058 | 0.01515 | 35301959～35826058 | 0.04545 | 35811485～35826058 | 0.04545 | *SPAG8、HINT2、FAM221B* |
|  | 3 | 131347165～131403096 | 0.04545 | 131347165～131403096 | 0.0303 | 131375701～131390472 | 0.0303 |  |
| **10** | 1 | 22839628～44285777 | 0.01515 | 27009334～27578854 | 0.01515 | 27326999～27356071 | 0.01515 |  |
|  | 2 | 91532432～99080246 | 0.01515 | 96602622～97921013 | 0.01515 | 96602622～96796861 | 0.01515 |  |
|  | 3 | 100184063～102750623 | 0.01515 | 101715899～102750623 | 0.01515 | 102104521～102296461 | 0.0303 |  |
| **11** | 1 | 49075605～56344843 | 0.01515 | 49075605～56344843 | 0.01515 | 55703247～55735857 | 0.12121 |  |
|  | 2 | 5423775～5461834 | 0.04545 | 5423775～5461834 | 0.04545 | 5423775～5424552 | 0.07576 |  |
|  | 3 | 1857173～3078536 | 0.01515 | 1961869～3078536 | 0.01515 | 2190951～2424541 | 0.01515 |  |
| **12** | 1 | 79611374～112915434 | 0.01515 | 104100617～112856954 | 0.01515 | 104324230～104345412 | 0.01515 |  |
|  | 2 | 31545381～52470979 | 0.01515 | 31545381～50358888 | 0.01515 | 48272895～48883020 | 0.01515 | *VDR、TMEM106C、COL2A1、LOC728114、SENP1、RPL37P19、LOC101927180、PFKM、ASB8、LOC100421855、LOC390311、LOC100533659、C12orf68、OR10AD1、H1FNT、ZNF641、OR5BK1P、OR5BT1P、OR5BJ1P、OR8S21P、OR8T1P、ANP32D、C12orf54、RPS10P20* |
|  | 3 | 10786757～11286379 | 0.01515 | 10786757～11286379 | 0.01515 | 10871667～10978402 | 0.01515 |  |
| **13** | 1 | 19625370～20039464 | 0.01515 | 19625370～20039464 | 0.01515 | 19751544～19775873 | 0.01515 |  |
|  | 2 | 113536132～113773159 | 0.01515 | 113536132～113773159 | 0.01515 | 113747135～113773159 | 0.01515 | *MCF2L、F7* |
|  | 3 | 99356612～100517028 | 0.01515 | 99356612～100517028 | 0.01515 | 99356612～99378566 | 0.01515 |  |
| **14** | 1 | 38681269～45711594 | 0.01515 | 38681269～45711594 | 0.01515 | 44975606～45606287 | 0.01515 |  |
|  | 2 | 64882380～68035879 | 0.01515 | 65253232～68035879 | 0.01515 | 67147816～67800316 | 0.01515 |  |
|  | 3 | 21829129～22322690 | 0.01515 | 21829129～22322690 | 0.04545 | 21829129～21831652 | 0.0303 |  |
| **15** | 1 | 42436599～48495508 | 0.04545 | 42436599～48495508 | 0.04545 | 42618531～42652099 | 0.0303 |  |
|  | 2 | 73541513～75503147 | 0.01515 | 73541513～75503147 | 0.01515 | 75498548～75503147 | 0.01515 |  |
|  | 3 | 91083353～91483520 | 0.01515 | 91083353～91483520 | 0.01515 | 91430436～91450524 | 0.01515 |  |
| **16** | 1 | 746914～1842209 | 0.04545 | 746914～1116169 | 0.01515 | 847743～855717 | 0.01515 |  |
|  | 2 | 88809773～90099348 | 0.01515 | 89764549～90099348 | 0.01515 | 89925556～89998957 | 0.01515 |  |
|  | 3 | 19085298～23711813 | 0.01515 | 19085298～27761040 | 0.01515 | 19085298～19132780 | 0.04545 |  |
| **17** | 1 | 73565262～80786261 | 0.01515 | 73816026～73922941 | 0.01515 | 73824121～73919538 | 0.01515 | *UNC13D、WBP2、TRIM47、TRIM65、MRPL38、FBF1* |
|  | 2 | 4693508～5991344 | 0.01515 | 4693508～5991344 | 0.01515 | 5326162～5336791 | 0.01515 | *RPAIN、C1QBP* |
|  | 3 | 39998141～41031695 | 0.01515 | 40842084～41031695 | 0.04545 | 40854534～41031695 | 0.01515 |  |
| **18** | 1 | 29126670～50432602 | 0.07576 |  |  |  |  |  |
|  | 2 | 213592～9888069 | 0.01515 | 213592～9888069 | 0.01515 | 691173～706558 | 0.0303 | *ENOSF1* |
|  | 3 | 56279025～58039276 | 0.01515 | 56279025～58039276 | 0.10606 | 57021738～57026361 | 0.01515 |  |
| **19** | 1 | 37726433～38899548 | 0.01515 | 37726433～38899548 | 0.01515 | 38847431～38897502 | 0.0303 |  |
|  | 2 | 19230868～21326358 | 0.01515 | 19230868～21326358 | 0.01515 | 19758190～20026163 | 0.01515 |  |
|  | 3 | 11327626～15490485 | 0.01515 | 13875626～14705509 | 0.04545 | 14139004～14165278 | 0.0303 | *RLN3、IL27RA、PALM3* |
| **20** | 1 | 3838441～17950545 | 0.01515 | 3838441～17950545 | 0.01515 | 10280083～10394046 | 0.0303 |  |
|  | 2 | 45242269～47993898 | 0.01515 | 47117176～47993898 | 0.01515 | 47615074～47682893 | 0.04545 |  |
|  | 3 | 37570725～43270105 | 0.01515 | 37570725～43270105 | 0.07576 |  |  |  |
| **21** | 1 | 42860485～46934861 | 0.01515 | 45220787～45564766 | 0.01515 | 45232322～45506819 | 0.01515 |  |
|  | 2 | 14798451～33058075 | 0.01515 | 15974409～33058075 | 0.04545 | 16340289～19165259 | 0.01515 | *NRIP1、LOC101927745、CYCSP42、RAD23BLP、USP25、RBPMSLP、LINC00478、VDAC2P1、RPS26P5、MIR99A、MIRLET7C、MIR125B2、LOC101927771、NEK4P1、C21orf37、TRNAG27、RPL39P40、CXADR、BTF3L4P1、BTG3、C21orf91-OT1、C21orf91* |
|  | 3 | 41516366～41725579 | 0.65152 | 41516366～41725579 | 0.13636 |  |  |  |
| **22** | 1 | 17978600～20977077 | 0.01515 | 17978600～20977077 | 0.01515 | 19048413～19183787 | 0.01515 |  |
|  | 2 | 30077361～32784051 | 0.01515 | 30864817～31090183 | 0.01515 | 30934816～30983971 | 0.01515 |  |
|  | 3 | 50313438～50618641 | 0.01515 | 50313438～50618641 | 0.01515 | 50313438～50320943 | 0.09091 |  |

Patients. III-15

| **chr** | **rank** | **eMSS** | **p-value** | **1st eMSS refinement** | **p-value** | **2nd eMSS refinement** | **p-value** | ***Gene symbol*** |
| --- | --- | --- | --- | --- | --- | --- | --- | --- |
| **1** | 1 | 998395～15419768 | 0.01515 | 8412989～14109667 | 0.01515 | 11141187～11273418 | 0.0303 | *EXOSC10、LOC101929136、MTOR、MTOR-AS1、ANGPTL7* |
|  | 2 | 117957288～162737116 | 0.01515 | 117957288～162737116 | 0.01515 | 146643555～146656097 | 0.01515 | *PRKAB2、LOC100505794、PDIA3P1、FMO5* |
|  | 3 | 35260929～43689679 | 0.01515 | 35260929～36316571 | 0.01515 | 35562965～35686521 | 0.01515 |  |
| **2** | 1 | 74332825～86015096 | 0.01515 | 79422965～86015096 | 0.01515 | 84834063～84861723 | 0.06061 |  |
|  | 2 | 135713703～141245204 | 0.01515 | 135713703～141245204 | 0.01515 | 135745722～136379194 | 0.01515 |  |
|  | 3 | 210382858～218941095 | 0.01515 | 216176807～218941095 | 0.01515 | 218712918～218770984 | 0.01515 | *TNS1* |
| **3** | 1 | 51990315～52867718 | 0.01515 | 51990315～52867288 | 0.01515 | 52453893～52475117 | 0.01515 |  |
|  | 2 | 9985656～38125591 | 0.01515 | 9985656～10167264 | 0.01515 | 10059658～10138189 | 0.01515 |  |
|  | 3 | 126365127～130360527 | 0.01515 | 126451937～129267910 | 0.01515 | 128755814～128890350 | 0.0303 |  |
| **4** | 1 | 103611845～123797428 | 0.10606 |  |  |  |  |  |
|  | 2 | 47322190～61529722 | 0.01515 | 52938243～61529722 | 0.10606 |  |  |  |
|  | 3 | 88767008～89648687 | 0.01515 | 88767008～89648687 | 0.04545 | 88959381～88997102 | 0.13636 |  |
| **5** | 1 | 93731985～112917278 | 0.01515 | 93731985～112917278 | 0.01515 | 107587767～108521893 | 0.01515 | *FBXL17、RPS20P3、LINC01023、FER、LOC100422561、*  *LOC100820733、GJA1P1* |
|  | 2 | 31317494～43245850 | 0.01515 | 37724990～38997045 | 0.01515 | 38823254～38950776 | 0.01515 |  |
|  | 3 | 178199558～179227154 | 0.01515 | 178199558～179227154 | 0.01515 | 178396742～178412667 | 0.01515 |  |
| **6** | 1 | 166827436～170713682 | 0.04545 | 169637911～170713682 | 0.01515 | 169637911～170176648 | 0.01515 | *THBS2、WDR27、LOC101929569、LOC101929543、*  *C6orf120、PHF10、TCTE3、ERMARD* |
|  | 2 | 15627553～24588884 | 0.01515 | 15627553～24588884 | 0.04545 | 24358483～24433693 | 0.01515 | *DCDC2、KAAG1、MRS2、GPLD1* |
|  | 3 | 30627216～30899746 | 0.01515 | 30627216～30899746 | 0.06061 |  |  |  |
| **7** | 1 | 90894102～128784963 | 0.01515 | 90894102～128784963 | 0.01515 | 98848752～99293358 | 0.01515 | *MYH16、ARPC1A、ARPC1B、PDAP1、BUD31、ATP5J2-PTCD1、PTCD1、CPSF4、LOC100131859、ATP5J2、TRNAW6、ZNF789、ZNF394、ZKSCAN5、LOC100419451、FAM200A、ZNF655、LOC101929496、LOC100289187、ZSCAN25、LOC442603、CYP3A5、CYP3A7-CYP3AP1、CYP3AP1* |
|  | 2 | 34889222～44580876 | 0.01515 | 36729757～44580876 | 0.01515 | 44553238～44580876 | 0.01515 | *NPC1L1* |
|  | 3 | 76751921～77256713 | 0.04545 | 76751921～77256713 | 0.01515 | 76984572～76991935 | 0.04545 | *GSAP* |
| **8** | 1 | 64321899～103357685 | 0.01515 | 64321899～71075113 | 0.01515 | 67411019～67793056 | 0.01515 |  |
|  | 2 | 21996617～25149501 | 0.01515 | 23186007～25149501 | 0.01515 | 23186007～23292985 | 0.0303 |  |
|  | 3 | 1830956～3253768 | 0.01515 | 1830956～2005675 | 0.01515 | 1953194～1953620 | 0.06061 |  |
| **9** | 1 | 91610543～99064425 | 0.01515 | 91610543～99064425 | 0.01515 | 95070095～95088255 | 0.01515 | *NOL8、CENPP* |
|  | 2 | 138905136～139959292 | 0.01515 | 139565150～139699034 | 0.04545 | 139616742～139649612 | 0.01515 | *FAM69B、SNHG7、SNORA43、SNORA17、LCN10、*  *LCN6、LOC100128593、LCN8* |
|  | 3 | 136031346～136132954 | 0.01515 | 136031346～136132954 | 0.04545 | 136031346～136101508 | 0.06061 |  |
| **10** | 1 | 70531217～84745256 | 0.01515 | 70531217～84745256 | 0.01515 | 80921864～81682448 | 0.01515 | *ZMIZ1、PPIF、ZCCHC24、TPRX1P1、LOC729815、EIF5AL1、RPS12P18、SFTPA2、MBL3P、SFTPA3P、SFTPA1、LOC101929525、LOC101929506、BEND3P3、LOC101060691、NUTM2B、LOC642361、NUTM2E、LOC100421010、NPAP1P2、CTSLP6、PGGT1BP2、LOC100288974、MBL1P* |
|  | 2 | 27009334～27529313 | 0.01515 | 27009334～27529313 | 0.01515 | 27326999～27381349 | 0.01515 |  |
|  | 3 | 121140321～121674228 | 0.01515 | 121140321～121674228 | 0.01515 | 121551581～121674228 | 0.01515 | *INPP5F、LOC100533676、MCMBP、SEC23IP* |
| **11** | 1 | 49075605～56380971 | 0.01515 | 49075605～56380971 | 0.01515 | 55703247～55735857 | 0.12121 |  |
|  | 2 | 13398129～19955407 | 0.01515 | 13398129～19955407 | 0.01515 | 18633971～18741260 | 0.01515 |  |
|  | 3 | 78673374～83810113 | 0.04545 | 78673374～83810113 | 0.01515 | 82443672～82444021 | 0.0303 | *FAM181B* |
| **12** | 1 | 97639144～133778796 | 0.01515 | 124274474～124826676 | 0.01515 | 124399155～124826676 | 0.01515 | *DNAH10、CCDC92、ZNF664、ZNF664-FAM101A、FAM101A、NCOR2* |
|  | 2 | 47178307～52713088 | 0.01515 | 47178307～50734582 | 0.01515 | 48272895～48884535 | 0.01515 | *VDR、TMEM106C、COL2A1、LOC728114、SENP1、RPL37P19、LOC101927180、PFKM、ASB8、LOC100421855、LOC390311、LOC100533659、C12orf68、OR10AD1、H1FNT、ZNF641、OR5BK1P、OR5BT1P、OR5BJ1P、OR8S21P、OR8T1P、ANP32D、C12orf54、RPS10P20* |
|  | 3 | 68720627～71898358 | 0.01515 | 68720627～71898358 | 0.01515 | 69980028～69995347 | 0.06061 |  |
| **13** | 1 | 46728924～52035344 | 0.07576 |  |  |  |  |  |
|  | 2 | 99356612～100517028 | 0.01515 | 99356612～100517028 | 0.01515 | 99356612～99378566 | 0.01515 |  |
|  | 3 | 107516667～110817171 | 0.01515 | 107516667～110817171 | 0.01515 | 110408608～110813532 | 0.0303 | *IRS2、RN7SKP10、LOC101927695、LOC101927646、LOC101927712、COL4A1* |
| **14** | 1 | 39627433～47426880 | 0.01515 | 39703324～47426880 | 0.04545 | 44976241～45606287 | 0.01515 |  |
|  | 2 | 21785790～22322690 | 0.01515 | 21785790～22322690 | 0.01515 | 21785790～21836400 | 0.07576 |  |
|  | 3 | 94936222～105173831 | 0.01515 | 94936222～100607595 | 0.01515 | 96781912～96795997 | 0.0303 |  |
| **15** | 1 | 73541513～100821576 | 0.01515 | 90455940～91578240 | 0.01515 | 91482935～91517780 | 0.01515 | *UNC45A、RCCD1、PRC1、PRC1-AS1* |
|  | 2 | 40072311～41222855 | 0.01515 | 40751555～41222855 | 0.04545 | 41149161～41222855 | 0.04545 |  |
|  | 3 | 64418397～66786706 | 0.01515 | 64418397～66786706 | 0.01515 | 64558040～64889463 | 0.01515 |  |
| **16** | 1 | 633354～1270111 | 0.01515 | 633354～1270111 | 0.01515 | 732287～746914 | 0.0303 | *STUB1、JMJD8、WDR24、FBXL16* |
|  | 2 | 89763263～90161034 | 0.01515 | 90067079～90161034 | 0.01515 | 90109711～90110857 | 0.0303 |  |
|  | 3 | 19680546～23080653 | 0.01515 | 22178256～23080653 | 0.04545 | 22269867～22391184 | 0.01515 |  |
| **17** | 1 | 19645938～34264862 | 0.01515 | 26691321～34264862 | 0.01515 | 29058863～29226228 | 0.01515 |  |
|  | 2 | 76870581～81006387 | 0.01515 | 76870581～78061872 | 0.01515 | 77704912～77758691 | 0.01515 | *ENPP7、CBX2* |
|  | 3 | 73816026～73914024 | 0.01515 | 73816026～73914024 | 0.07576 |  |  |  |
| **18** | 1 | 43262501～44272090 | 0.01515 | 43262501～44272090 | 0.01515 | 43547127～43579395 | 0.01515 | *EPG5、PSTPIP2* |
|  | 2 | 6999628～7567803 | 0.01515 | 6999628～7567803 | 0.01515 | 7002412～7008583 | 0.01515 |  |
|  | 3 | 25573622～28728411 | 0.01515 | 25573622～28728411 | 0.01515 | 28719674～28728411 | 0.04545 | *LOC101927698、DSC1* |
| **19** | 1 | 37677748～39294466 | 0.01515 | 38709568～39106924 | 0.01515 | 38996623～38998497 | 0.07576 |  |
|  | 2 | 11459260～15230219 | 0.01515 | 11465316～12996740 | 0.01515 | 12551715～12989560 | 0.01515 |  |
|  | 3 | 19735257～21720803 | 0.01515 | 19735257～21720803 | 0.01515 | 21544571～21609644 | 0.01515 |  |
| **20** | 1 | 34208516～47361725 | 0.01515 | 34208516～47361725 | 0.01515 | 44038574～44259673 | 0.04545 |  |
|  | 2 | 61834695～62850376 | 0.01515 | 61873080～62173925 | 0.07576 |  |  |  |
|  | 3 | 4705865～4721688 | 0.10606 |  |  |  |  |  |
| **21** | 1 | 45770264～47571845 | 0.01515 | 45770264～46228528 | 0.01515 | 46000398～46021954 | 0.01515 |  |
|  | 2 | 30250615～35893737 | 0.01515 | 30250615～35893737 | 0.01515 | 35472989～35473518 | 0.01515 |  |
|  | 3 | 16334963～18976385 | 0.01515 | 16334963～18976385 | 0.07576 |  |  |  |
| **22** | 1 | 19126631～22318480 | 0.01515 | 19126631～22318480 | 0.01515 | 20755098～20847472 | 0.01515 |  |
|  | 2 | 30077361～31153162 | 0.01515 | 30077361～30921371 | 0.04545 | 30864817～30891859 | 0.19697 |  |
|  | 3 | 50303533～50515843 | 0.01515 | 50303533～50321137 | 0.10606 |  |  |  |
