# Supplementary material for: Maximal Segmental Score Method for Localizing Recessive Disease Variants Based on Sequence Data
Source: Front Genet. 2020 Jun 12;11:555. doi: 10.3389/fgene.2020.00555 (PMC7325894; doi:10.3389/fgene.2020.00555)
Supplement: Supplementary file 1 [file Presentation_1.zip › Figure S3.DOCX]

Supplementary Table S3. Ranking of known pathogenic variants in each of the four patients used in the HDR-del method.

|  |  |  | Pathogenic region  HDR-del (ROHs at least 1 Mb long) | | | | |  | HDR-del  (ROHs in range + 1.5 Mb)^7^ | |  | | HDR-del  (ROHs in range + 0.5 Mb)^8^ | |
| --- | --- | --- | --- | --- | --- | --- | --- | --- | --- | --- | --- | --- | --- | --- |
| Patient | Disease | Chr | Test stat | *P* value | Position  (range) | Num^1^ | Rank^4^ |  | Num^2^ | Rank^5^ |  | | Num^3^ | Rank^6^ |
| OI | OI | 8 | 8.5080 | 0.0303 | 21,471,941-23,622,382  (2.15 Mb) | 375 | 32 |  | 345 | 26 |  | 110 | | 7 |
| F1 | MIA | 2 | 4.4981 | 0.0606 | 45,839,387-47,882,706  (2.04 Mb) | 223 | 26 |  | 209 | 25 |  | 79 | | 8 |
| F4 | MIA | 2 | 5.6224 | 0.1212 | 43,413,118-54,587,596  (11.17 Mb) | 205 | 26 |  | 2 | 1 |  | 1 | | 1 |
| F6 | MIA | 2 | 12.2375 | 0.0303 | 45,171,842-52,799,698  (7.63 Mb) | 213 | 7 |  | 4 | 2 |  | 2 | | 1 |

Abbreviations: OI, Osteogenesis Imperfecta; MIA, Multiple Intestinal Atresia; Chr, chromosome; Test stat, t-statistic; Num^1^: Numbers of candidate regions in HDR-del; Num^2^: Numbers of candidate regions, including the range + 1.5 Mb of the corresponding pathogenic region were used in HDR-del refinement; Num^3^: Numbers of candidate regions, including the range + 0.5 Mb of the corresponding pathogenic region were used in HDR-del refinement; Rank^4^, order of test statistic for pathogenic variant among the numbers of candidate regions in HDR-del (Num^1^); Rank^5^, order of test statistic for pathogenic variant among the Num^2^; Rank^6^, order of test statistic for pathogenic variant among the Num^3^. ROHs in range + 1.5 Mb^7^, ROHs of length between + 1.5 Mb of the lengths of the pathogenic regions. ROHs in range + 0.5 Mb^8^, ROHs of length between + 0.5 Mb of the lengths of the pathogenic regions
