# Supplementary material for: Maximal Segmental Score Method for Localizing Recessive Disease Variants Based on Sequence Data
Source: Front Genet. 2020 Jun 12;11:555. doi: 10.3389/fgene.2020.00555 (PMC7325894; doi:10.3389/fgene.2020.00555)
Supplement: Supplementary file 1 [file Presentation_1.zip › Figure S4.DOCX]

Supplementary Table S4. Ranking of known pathogenic variants in each of the two patients used in the HDR-del method.

|  |  |  | Pathogenic region  HDR-del (ROHs at least 1 Mb long) | | | | |  | HDR-del  (ROHs in range + 1.5 Mb)^7^ | |  | | HDR-del  (ROHs in range + 0.5 Mb)^8^ | |
| --- | --- | --- | --- | --- | --- | --- | --- | --- | --- | --- | --- | --- | --- | --- |
| Patient | Disease | Chr | Test stat | *P* value | Position  (range) | Num^1^ | Rank^4^ |  | Num^2^ | Rank^5^ |  | | Num^3^ | Rank^6^ |
| III-5 | OI | 12 | 19.0252 | 0.0303 | 34,175,508-52,404,618  (18.23 Mb) | 103 | 5 |  | 2 | 1 |  | 2 | | 1 |
| III-15 | OI | 12 | 14.8963 | 0.0303 | 45,410,075-52,602,013  (7.19 Mb) | 122 | 16 |  | 11 | 1 |  | 4 | | 1 |

Abbreviations: OI, Osteogenesis Imperfecta; MIA, Multiple Intestinal Atresia; Chr, chromosome; Test stat, t-statistic; Num^1^: Numbers of candidate regions in HDR-del; Num^2^: Numbers of candidate regions, including the range + 1.5 Mb of the corresponding pathogenic region were used in HDR-del refinement; Num^3^: Numbers of candidate regions, including the range + 0.5 Mb of the corresponding pathogenic region were used in HDR-del refinement; Rank^4^, order of test statistic for pathogenic variant among the numbers of candidate regions in HDR-del (Num^1^); Rank^5^, order of test statistic for pathogenic variant among the Num^2^; Rank^6^, order of test statistic for pathogenic variant among the Num^3^. ROHs in range + 1.5 Mb^7^, ROHs of length between + 1.5 Mb of the lengths of the pathogenic regions. ROHs in range + 0.5 Mb^8^, ROHs of length between + 0.5 Mb of the lengths of the pathogenic regions
