# Supplementary material for: Maximal Segmental Score Method for Localizing Recessive Disease Variants Based on Sequence Data
Source: Front Genet. 2020 Jun 12;11:555. doi: 10.3389/fgene.2020.00555 (PMC7325894; doi:10.3389/fgene.2020.00555)
Supplement: Supplementary file 1 [file Presentation_1.zip › Figure S5.DOCX]

Supplementary Table S5. Power and type I error comparison with differing scenarios in our eMSS calculations under one sample t test.

| Power (Type I error) | Number of haplotype blocks | |
| --- | --- | --- |
| Haplotype frequency | moderate density  36 Blocks (including 110 SNPs) | low density  15 Blocks (including 47 SNPs) |
| extreme | 31.5% (9%) | 55.1% (2.4%) |
| non-extreme | 5.5 % (11.5%) | 11.3% (6.9%) |
