# Supplementary material for: Maximal Segmental Score Method for Localizing Recessive Disease Variants Based on Sequence Data
Source: Front Genet. 2020 Jun 12;11:555. doi: 10.3389/fgene.2020.00555 (PMC7325894; doi:10.3389/fgene.2020.00555)
Supplement: Supplementary file 1 [file Presentation_1.zip › Figure S6.DOCX]

Supplementary Table S6. Power and type I error comparison with differing numbers of cases and controls in our eMSS calculations under the scenario with moderate density, non-extreme haplotype frequency and AF=0.001.

| Simulation scenario | Moderate density, non-extreme haplotype frequency and AF=0.001^1^ | | |
| --- | --- | --- | --- |
| Numbers of cases and controls | 1 case vs 32 controls | 1 case vs 50 controls | 3 case vs 32 controls |
| Power (Type I error) | 65.3% (3.0%) | 66% (5.3%) | 86% (3.4%) |

^1^ The simulation scenario with moderate density haplotype block (36 Blocks, 110 SNPs), the frequency of one major haplotype was < 0.6 and the allele frequency of one causal variant=0.001.
