# Supplementary material for: Maximal Segmental Score Method for Localizing Recessive Disease Variants Based on Sequence Data
Source: Front Genet. 2020 Jun 12;11:555. doi: 10.3389/fgene.2020.00555 (PMC7325894; doi:10.3389/fgene.2020.00555)

**Supplementary Figure S1.** Autosomal recessive disease, one Osteogenesis imperfecta (OI) case and 32 ALSPAC controls. 5,628 SNPs on Chromosome 8.

**S1a. eMSS**


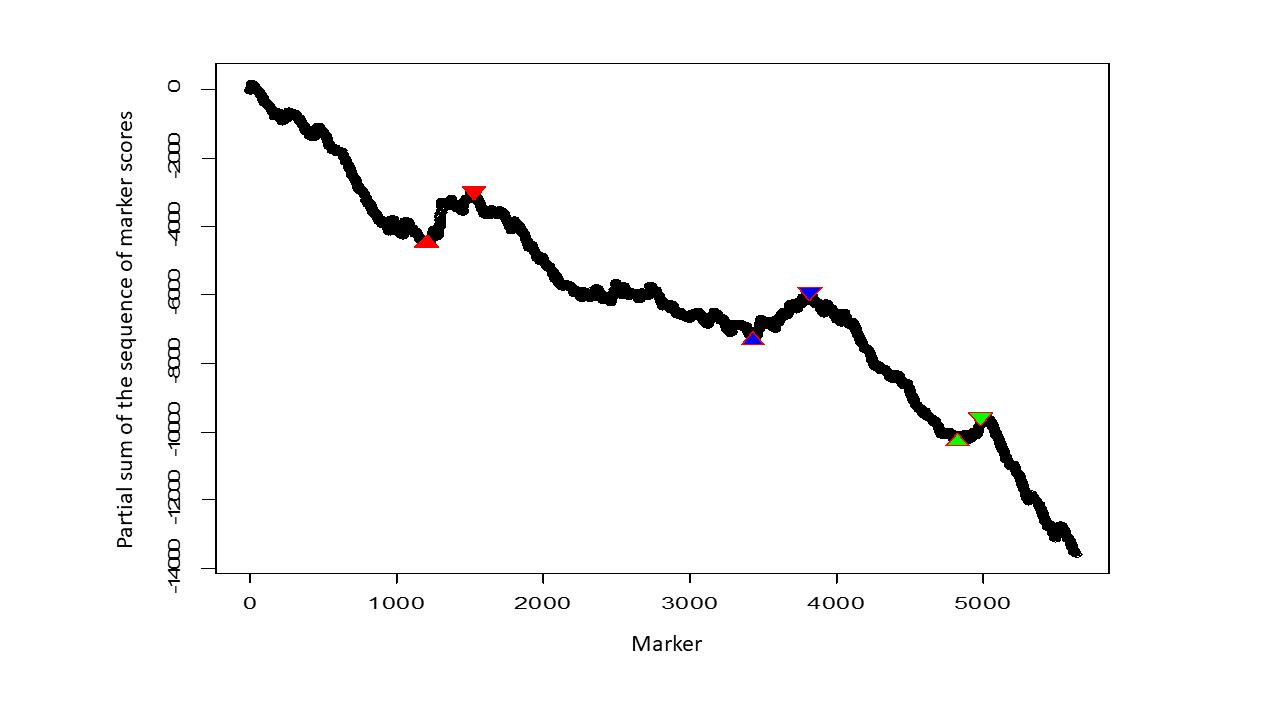


**S1b. eMSS refinement**


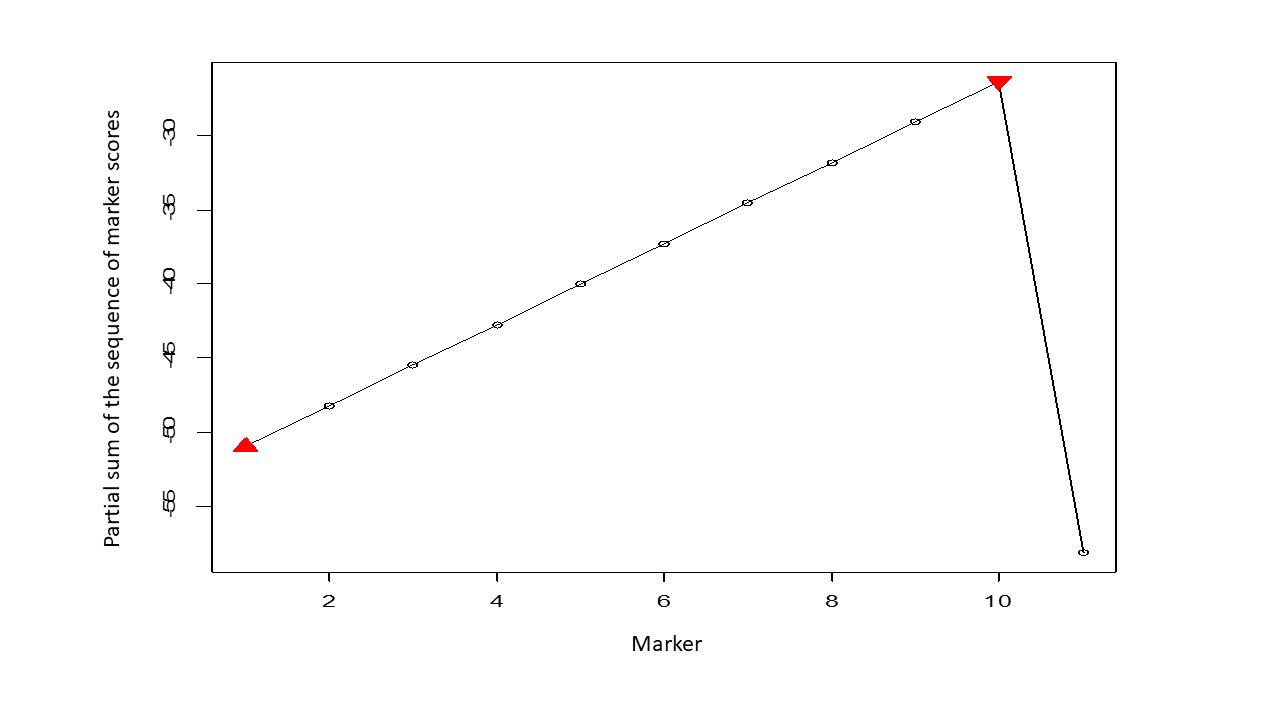


**Supplementary Figure S2.** Autosomal recessive disease, three Multiple Intestinal Atresia (MIA) cases and 32 ALSPAC controls. 3,484 SNPs on Chromosome 2.

**S2a. eMSS**


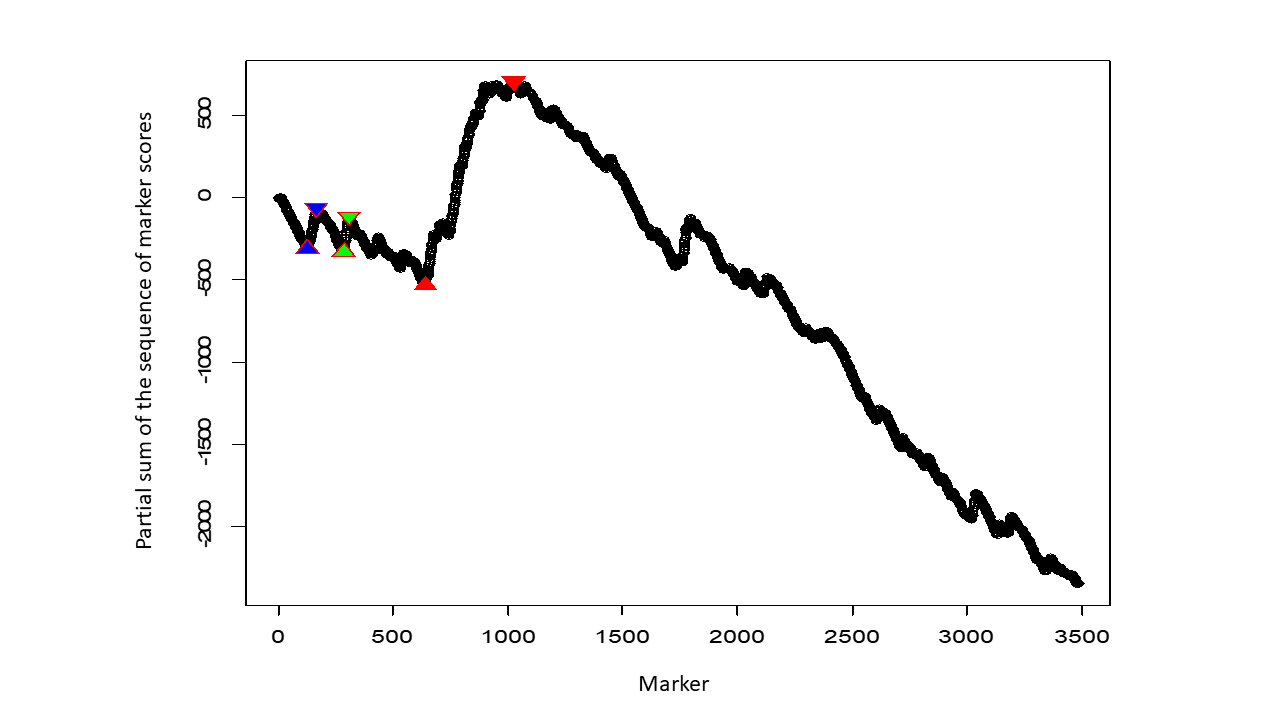

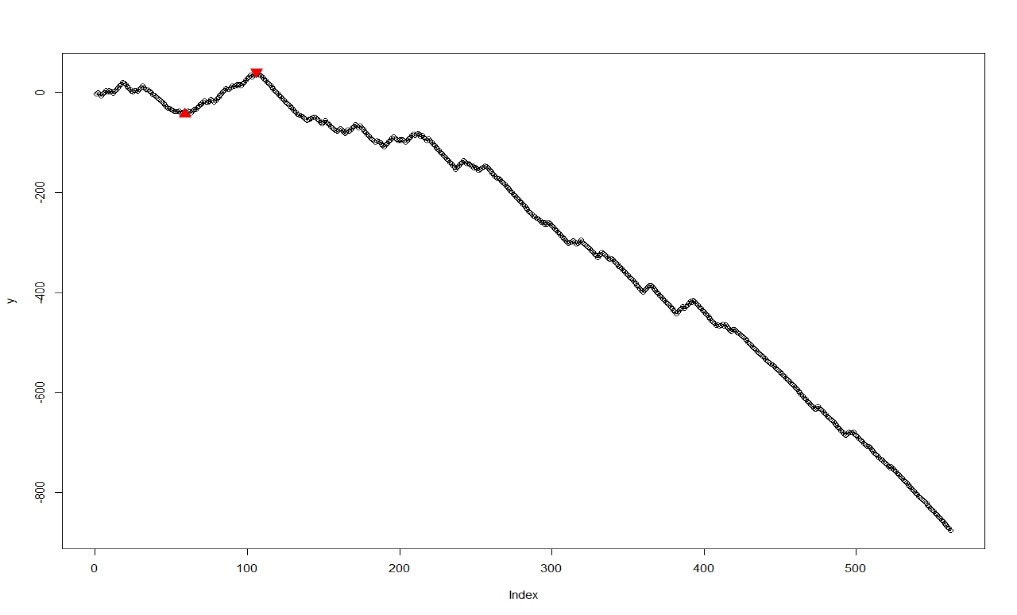


**S2b.eMSS refinement**


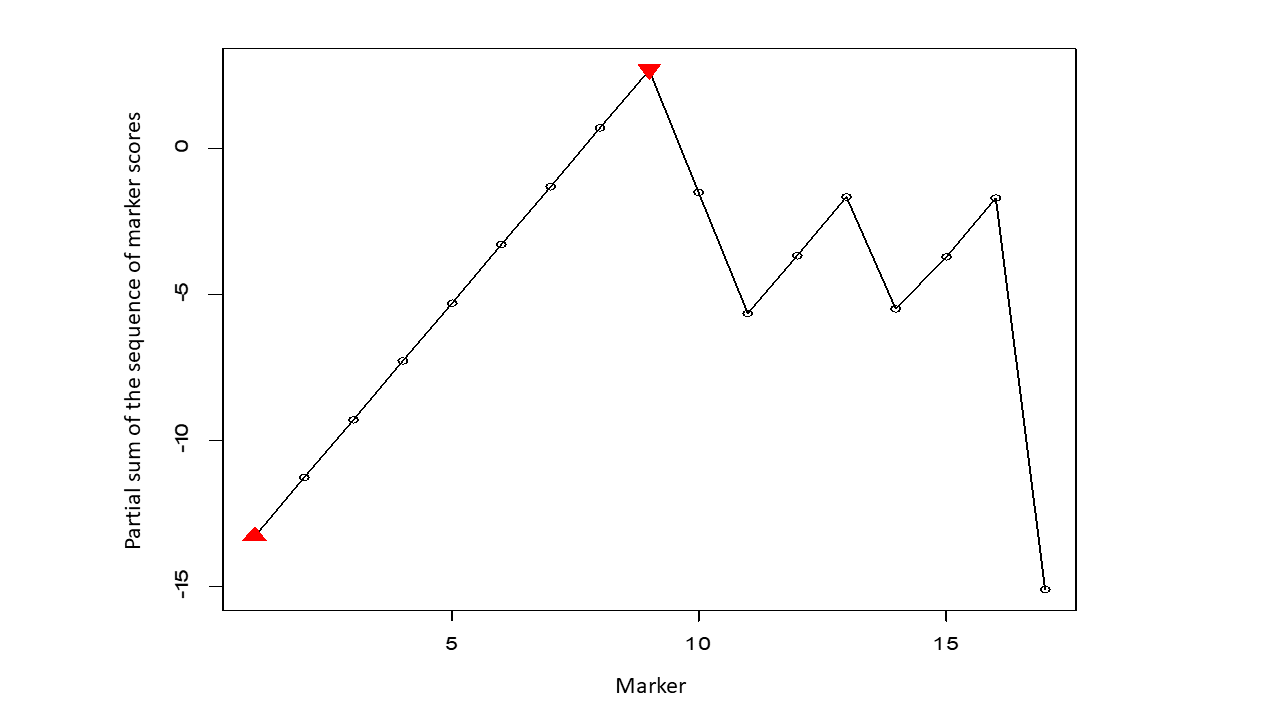


**Supplementary Figure S3.** Autosomal recessive disease, the MIA patient F1 and 32 ALSPAC controls. 8,465 SNPs on Chromosome 2.

**S3a. eMSS**


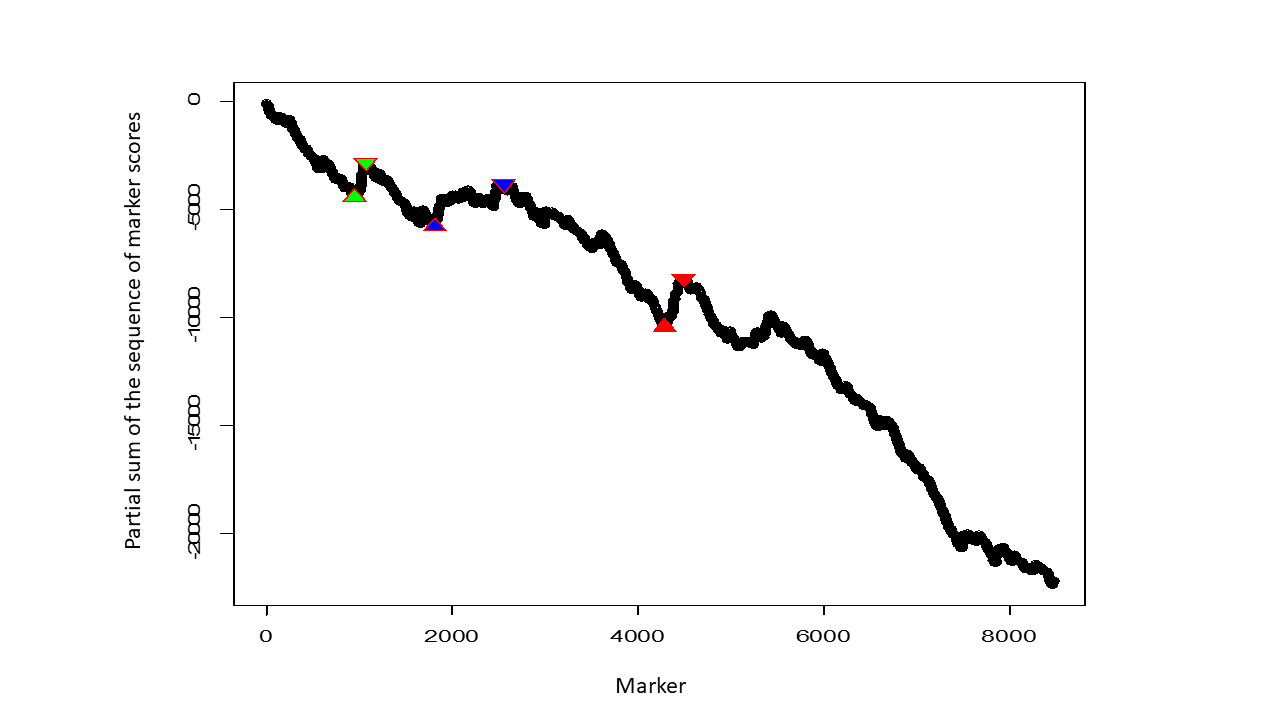


**S3b. eMSS refinement**


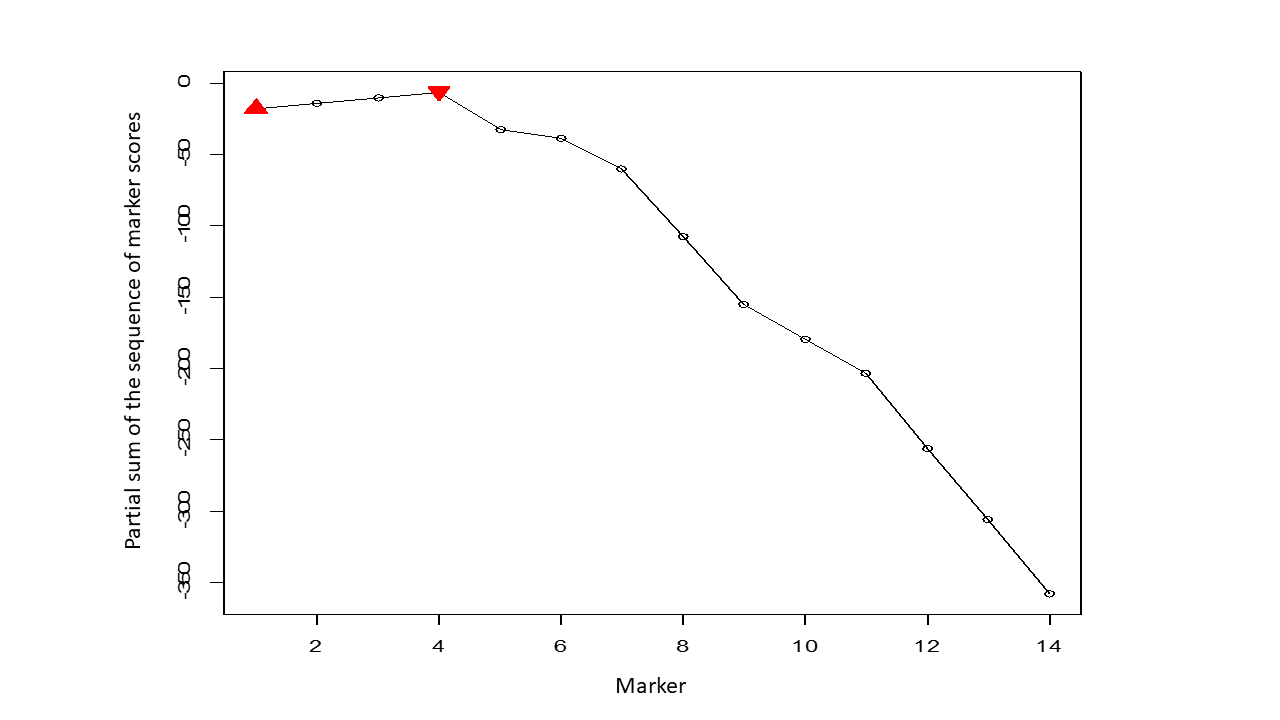


**Supplementary Figure S4.** Autosomal recessive disease, the MIA patient F4 and 32 ALSPAC controls. 7,380 SNPs on Chromosome 2.

**S4a. eMSS**


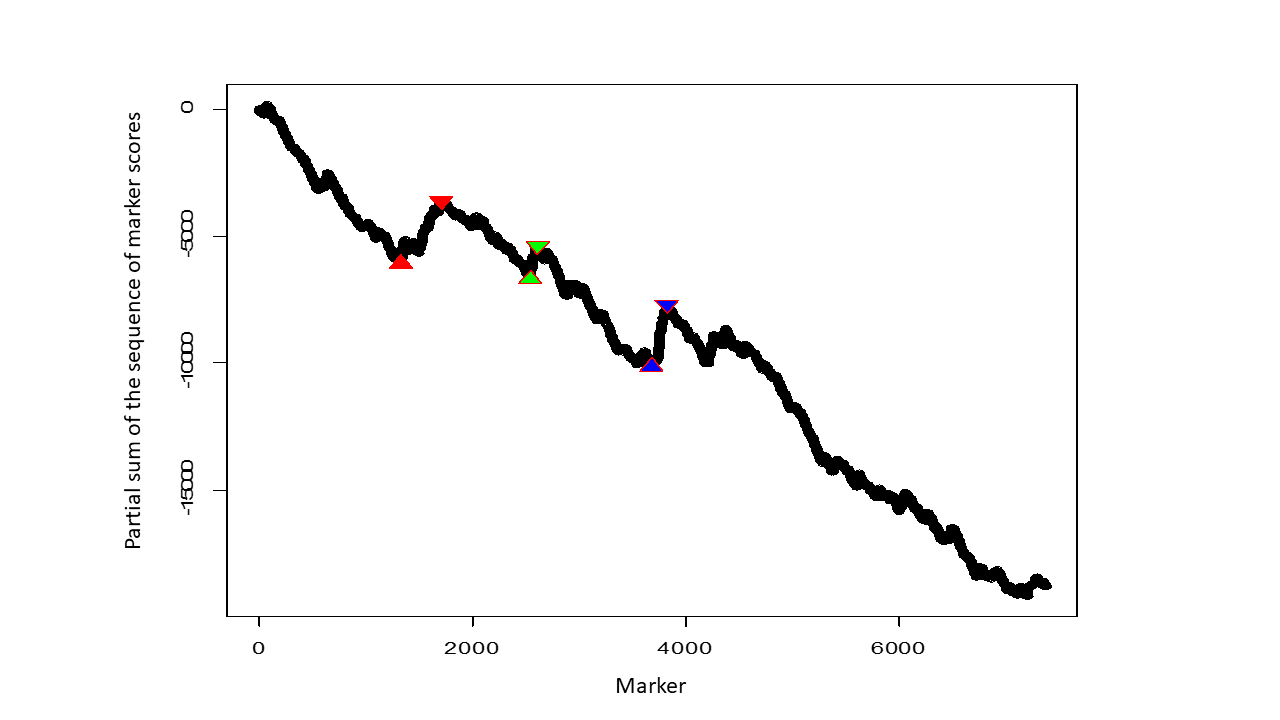


**S4b. eMSS refinement**


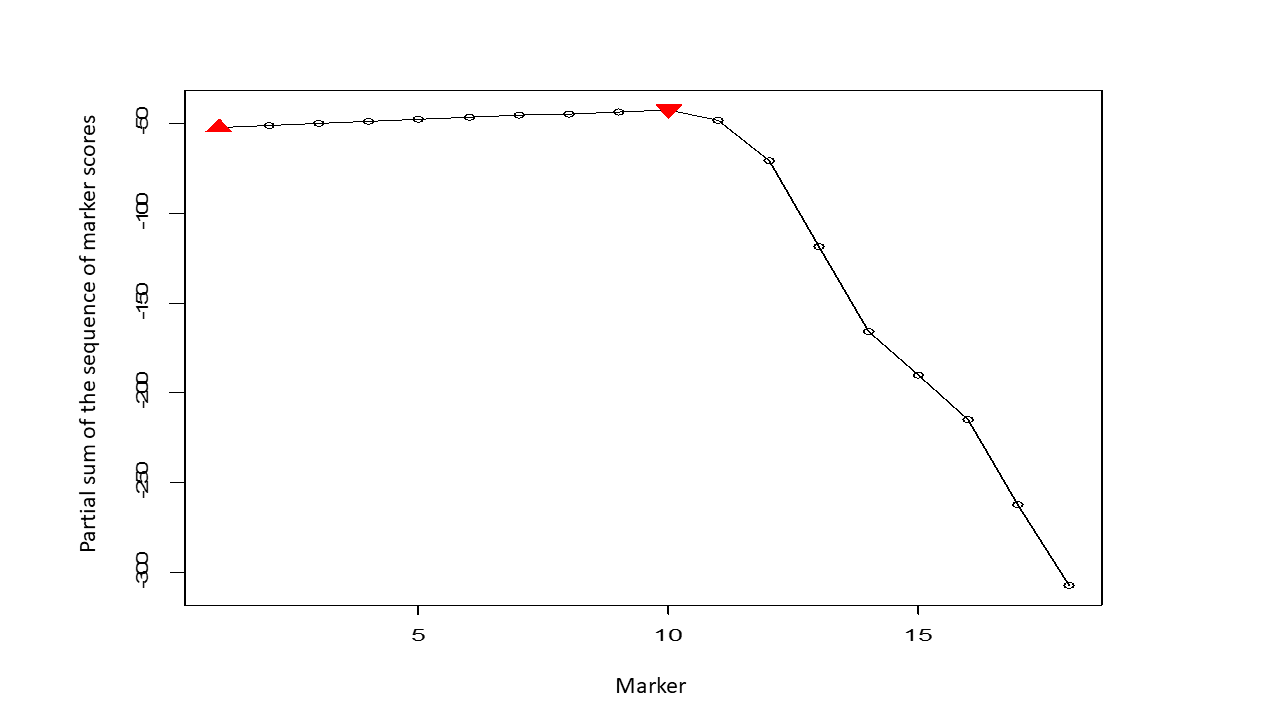


**Supplementary Figure S5.** Autosomal recessive disease, the MIA patient F6 and 32 ALSPAC controls. 7,562 SNPs on Chromosome 2.

**S5a. eMSS**


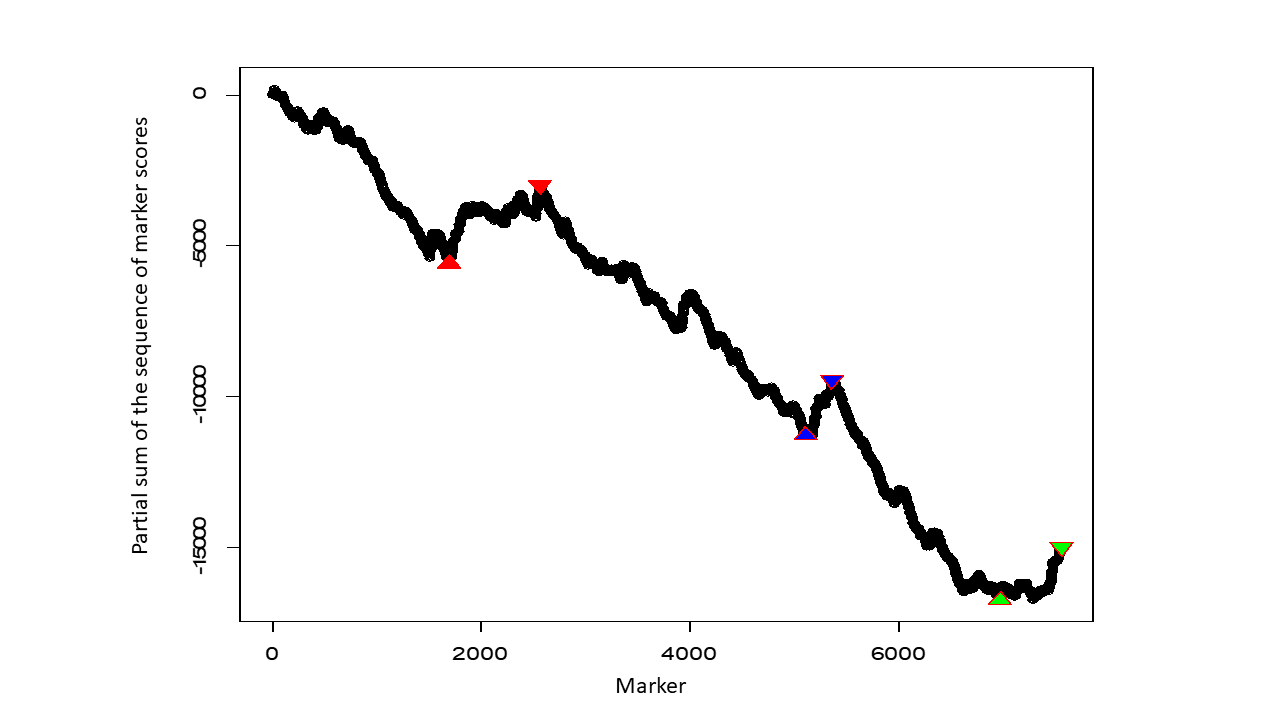


**S5b eMSS refinement**


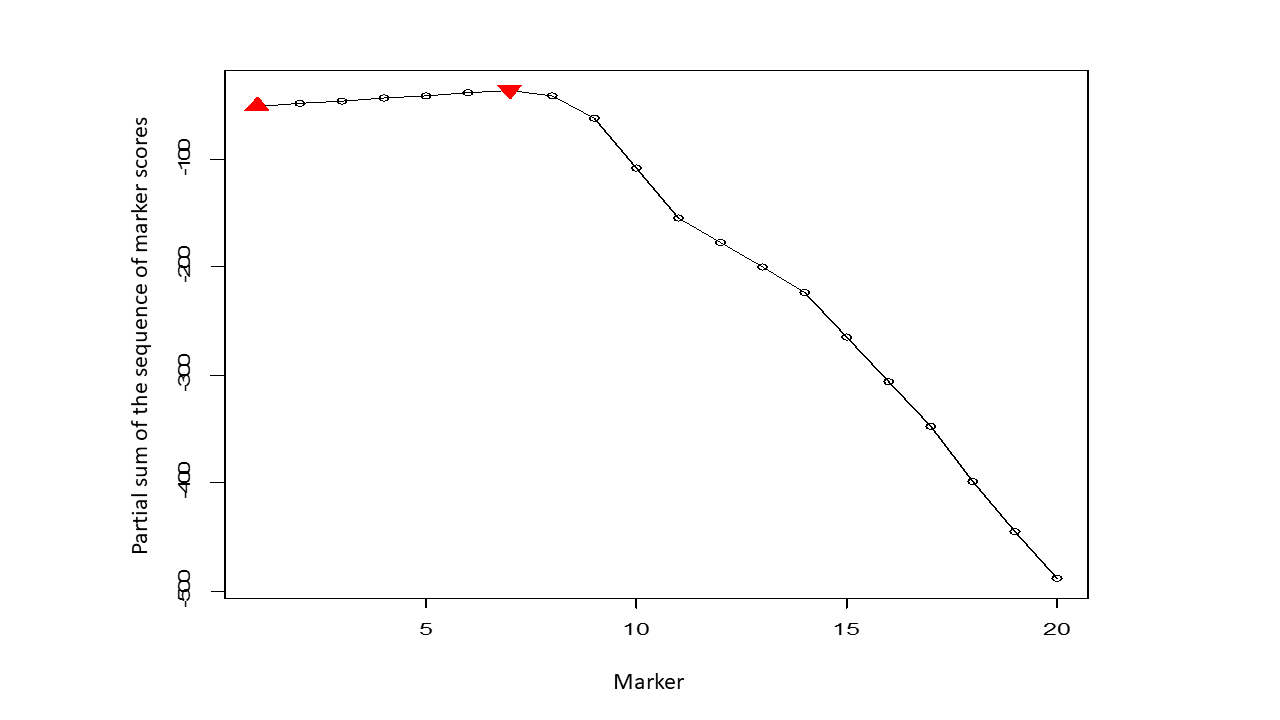


**Supplementary Figure S6.** Autosomal recessive disease, the OI Patient III-5 and 32 ALSPAC controls. 2,229 SNPs on Chromosome 12.

**S6a. Emss**

**
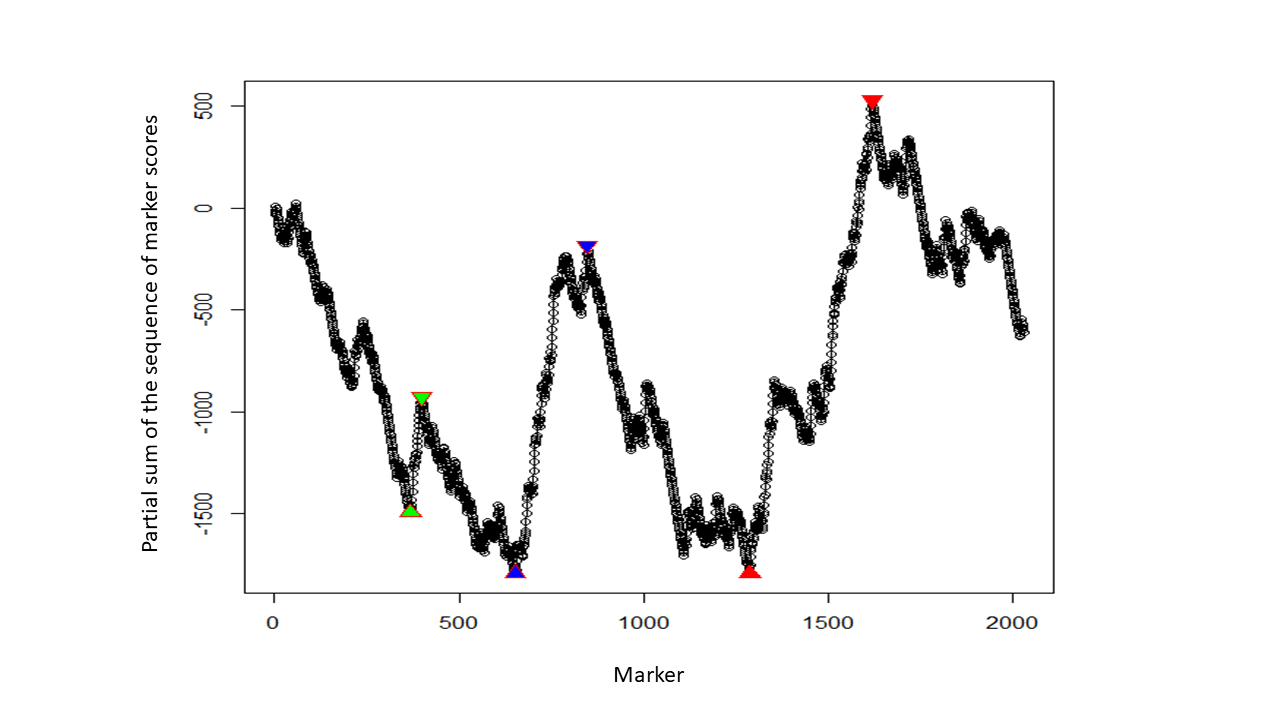
**

**S6b. eMSS**


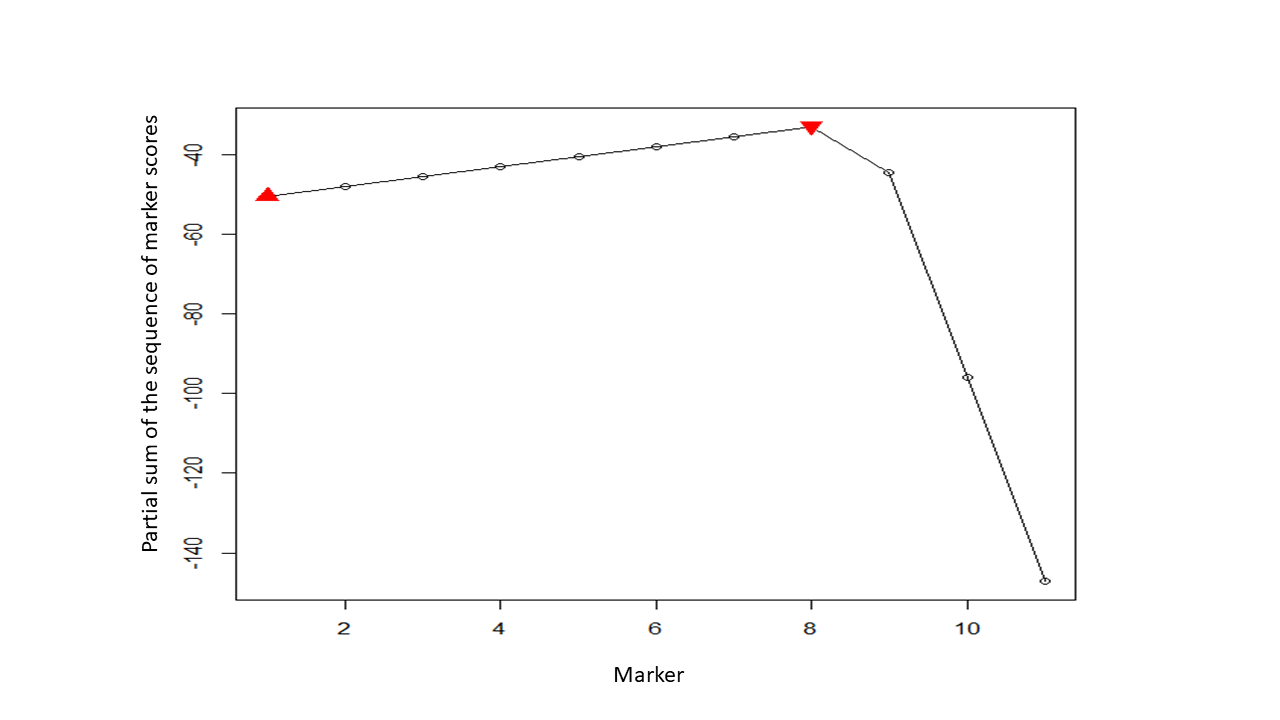


**Supplementary Figure S7.** Autosomal recessive disease, the OI Patient III-15 and 32 ALSPAC controls. 2,022 SNPs on Chromosome 12.

**S7a. eMSS**

**
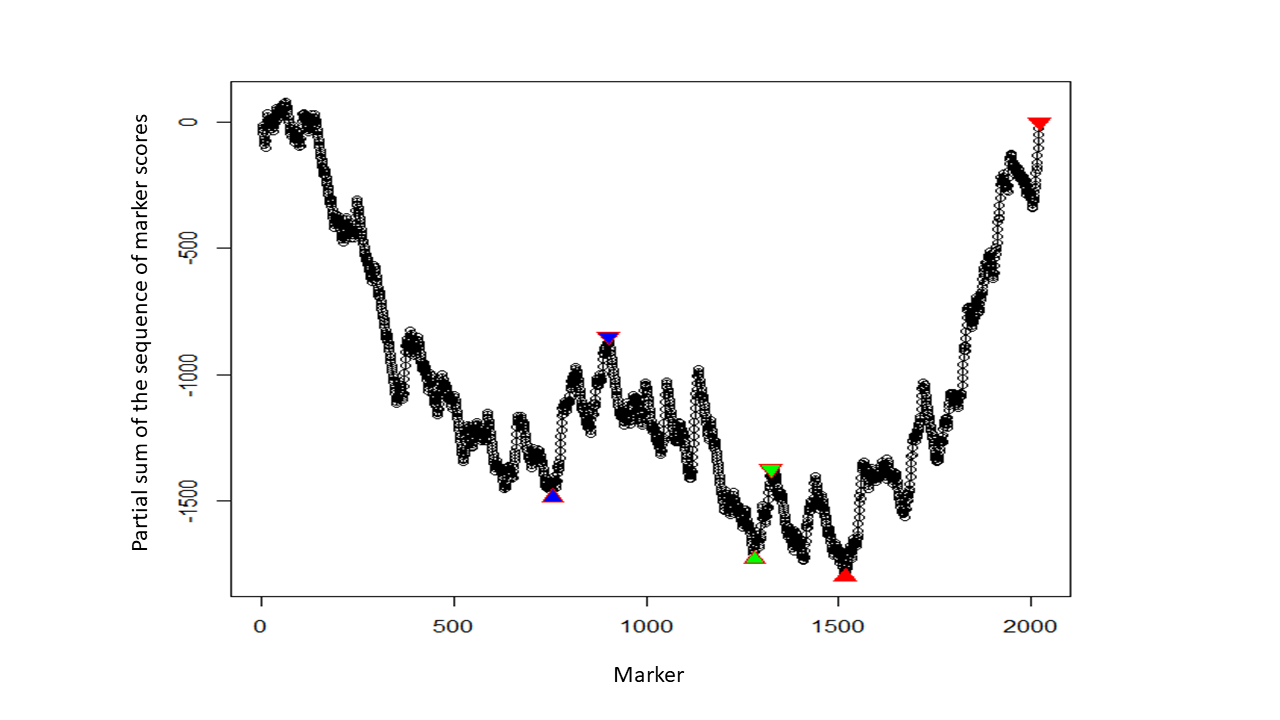
**

**S7b. eMSS**


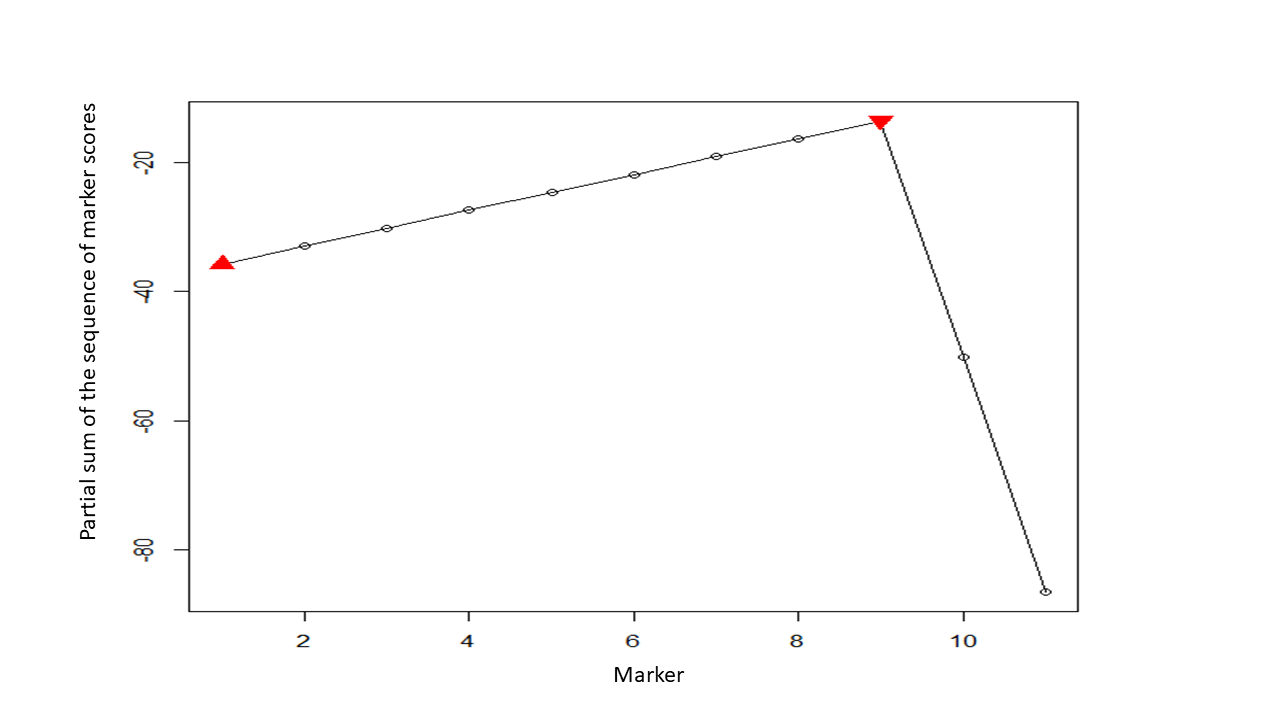

Supplement: Supplementary file 1 [file Presentation_1.zip › Figure S7.DOCX]
