# Supplementary material for: Maximal Segmental Score Method for Localizing Recessive Disease Variants Based on Sequence Data
Source: Front Genet. 2020 Jun 12;11:555. doi: 10.3389/fgene.2020.00555 (PMC7325894; doi:10.3389/fgene.2020.00555)

**Supplementary Figure S8.** **Illustration of MSS plot under different *f* using experimental data**: (a) *f*=1, useless wide MSS region, (b) *f*=1.25, no significant MSS region, and (c) *f*=1.14, legitimate MSS region for susceptible markers.

(a)


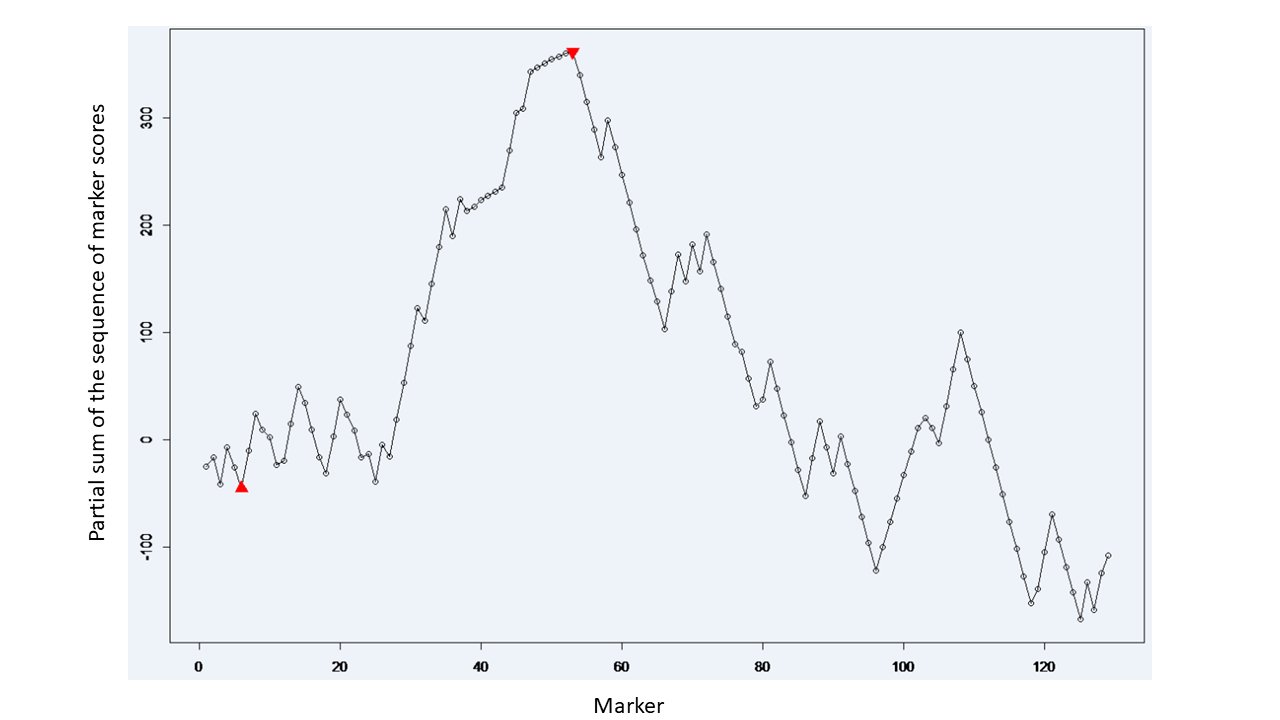


(b)
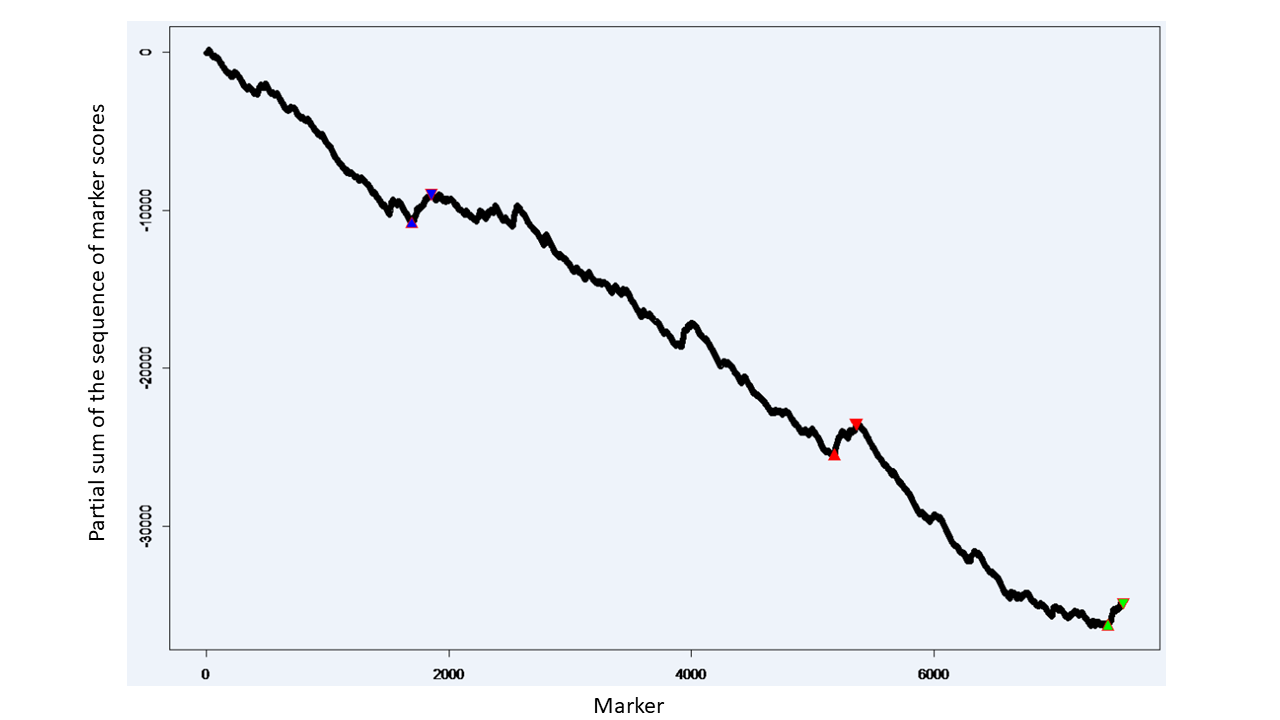


(c)
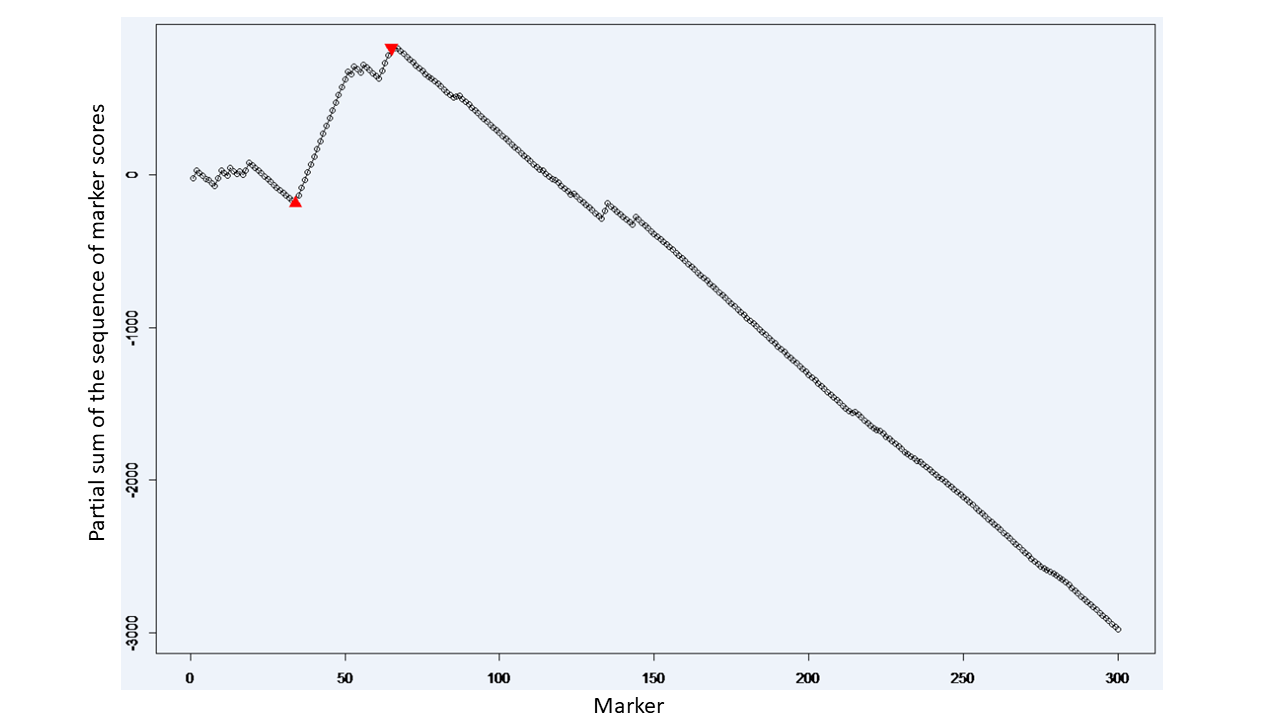

Supplement: Supplementary file 1 [file Presentation_1.zip › Figure S8.DOCX]
