# Supplementary material for: Maximal Segmental Score Method for Localizing Recessive Disease Variants Based on Sequence Data
Source: Front Genet. 2020 Jun 12;11:555. doi: 10.3389/fgene.2020.00555 (PMC7325894; doi:10.3389/fgene.2020.00555)
Supplement: Supplementary file 1 [file Presentation_1.zip › Figure S9.DOCX]

Supplementary Table S7. Power comparison with differing haplotype frequency and AF in HomozygosityMapper calculations under the scenario with high density 100 Blocks.

| **Power** | **high density^4^**  **100 Blocks (including 302 SNPs)** | |
| --- | --- | --- |
| **Haplotype frequency** | AF^3^=0.01 | AF=0.001 |
| extreme^1^ | 44% | 44% |
| non-extreme^2^ | 40% | 50% |

^1^ The frequency of one major haplotype was ≥ 0.8. ^2^ the frequency of one major haplotype was < 0.6.

^3^The allele frequency of one causal variant. ^4^ Homozygosity Mapper is suitable for longer sequence, so Homozygosity Mapper is not applicable for the other scenarios.
